# A network medicine approach to study comorbidities in heart failure with preserved ejection fraction

**DOI:** 10.1186/s12916-023-02922-7

**Published:** 2023-07-24

**Authors:** Jan D. Lanzer, Alberto Valdeolivas, Mark Pepin, Hauke Hund, Johannes Backs, Norbert Frey, Hans-Christoph Friederich, Jobst-Hendrik Schultz, Julio Saez-Rodriguez, Rebecca T. Levinson

**Affiliations:** 1 Institute for Computational Biomedicine, Heidelberg University, Faculty of Medicine, and Heidelberg University Hospital, Bioquant, Heidelberg, Germany; 2 Department of General Internal Medicine and Psychosomatics, Heidelberg University Hospital, Heidelberg, Germany; 3 Faculty of Biosciences, Heidelberg University, Heidelberg, Germany; 4 Informatics for Life, Heidelberg, Germany; 5 Roche Pharma Research and Early Development, Pharmaceutical Sciences, Roche Innovation Center Basel, Basel, Switzerland; 6 Institute of Experimental Cardiology, Medical Faculty Heidelberg, Heidelberg University, Heidelberg, Germany; 7 DZHK (German Centre for Cardiovascular Research), Partner Site Heidelberg/Mannheim, Im Neuenheimer Feld 669, 69120 Heidelberg, Germany; 8 Department of Cardiology, Internal Medicine III, Heidelberg University Hospital, Heidelberg, Germany

**Keywords:** HFpEF, Comorbidities, Network medicine, Comorbidity network, Disease-gene prediction

## Abstract

**Background:**

Comorbidities are expected to impact the pathophysiology of heart failure (HF) with preserved ejection fraction (HFpEF). However, comorbidity profiles are usually reduced to a few comorbid disorders. Systems medicine approaches can model phenome-wide comorbidity profiles to improve our understanding of HFpEF and infer associated genetic profiles.

**Methods:**

We retrospectively explored 569 comorbidities in 29,047 HF patients, including 8062 HFpEF and 6585 HF with reduced ejection fraction (HFrEF) patients from a German university hospital. We assessed differences in comorbidity profiles between HF subtypes via multiple correspondence analysis. Then, we used machine learning classifiers to identify distinctive comorbidity profiles of HFpEF and HFrEF patients. Moreover, we built a comorbidity network (HFnet) to identify the main disease clusters that summarized the phenome-wide comorbidity. Lastly, we predicted novel gene candidates for HFpEF by linking the HFnet to a multilayer gene network, integrating multiple databases. To corroborate HFpEF candidate genes, we collected transcriptomic data in a murine HFpEF model. We compared predicted genes with the murine disease signature as well as with the literature.

**Results:**

We found a high degree of variance between the comorbidity profiles of HFpEF and HFrEF, while each was more similar to HFmrEF. The comorbidities present in HFpEF patients were more diverse than those in HFrEF and included neoplastic, osteologic and rheumatoid disorders. Disease communities in the HFnet captured important comorbidity concepts of HF patients which could be assigned to HF subtypes, age groups, and sex. Based on the HFpEF comorbidity profile, we predicted and recovered gene candidates, including genes involved in fibrosis (*COL3A1*, *LOX,* SMAD9, PTHL), hypertrophy (GATA5, MYH7), oxidative stress (*NOS1*, *GSST1*, *XDH*), and endoplasmic reticulum stress (*ATF6*). Finally, predicted genes were significantly overrepresented in the murine transcriptomic disease signature providing additional plausibility for their relevance.

**Conclusions:**

We applied systems medicine concepts to analyze comorbidity profiles in a HF patient cohort. We were able to identify disease clusters that helped to characterize HF patients. We derived a distinct comorbidity profile for HFpEF, which was leveraged to suggest novel candidate genes via network propagation. The identification of distinctive comorbidity profiles and candidate genes from routine clinical data provides insights that may be leveraged to improve diagnosis and identify treatment targets for HFpEF patients.

**Graphical Abstract:**

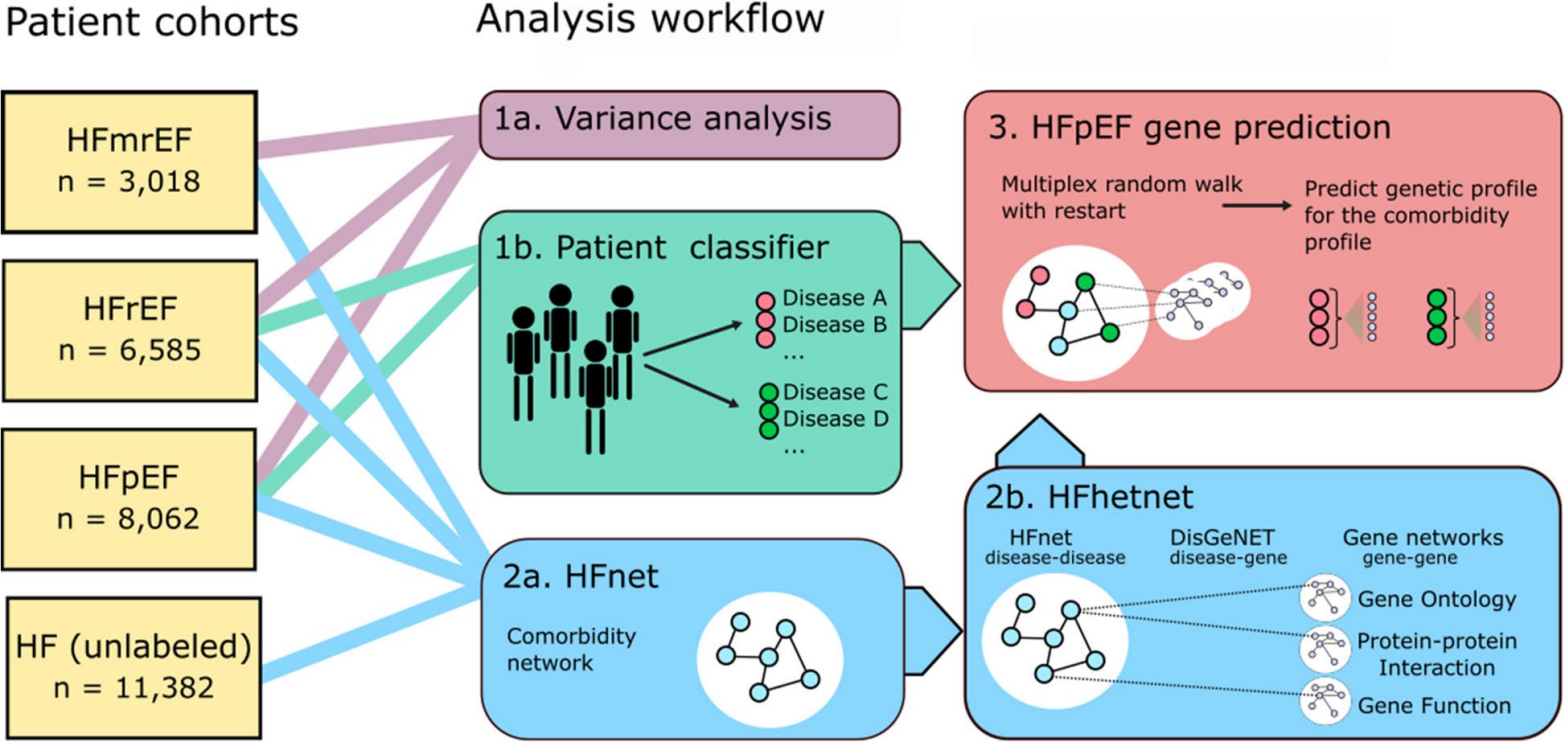

**Supplementary Information:**

The online version contains supplementary material available at 10.1186/s12916-023-02922-7.

## Background

Heart failure (HF) with preserved ejection fraction (HFpEF) represents an unmet public health concern with rising prevalence [1, 2]. Accumulating evidence indicates HFpEF is clinically and biologically distinct from HF with reduced ejection fraction (HFrEF), as reflected by missing therapy response in HFpEF patients to drugs effective in HFrEF [3]. HF patients suffer from a wide range of comorbidities, which are considered important for HF development and progression [4]. In the pathogenesis of HFpEF, comorbidities have been suggested as causal factors [3, 5] and could possibly be linked to genetic etiology. Treatment of comorbidity has also been shown to have beneficial effects of cardiac physiology [6], emphasizing the potential to address HF subtypes through their comorbidities.

Systems medicine attempts to model disease in a holistic manner. One facet of this, network medicine, is used to analyze complex systems such as patients, organs, or cells via network representation [7, 8]. Comorbidity networks represent diseases as nodes, connected via edges based on co-occurrence in patients. These networks can be used to define disease modules or explore topological changes between patient cohorts [9–12]. Previous work has shown that disease comorbidity is also often linked to shared disease genes that locate close together in gene-based networks like protein–protein interaction networks [12, 13]. This observation is often the basis of network-based gene prediction, where novel disease genes are predicted based on network proximity to known disease genes.

Cardiovascular diseases are particularly suited for system medicine approaches due to the typical multiorgan involvement [14] and multifactorial etiology [15]. To date, such approaches to study HFpEF have been limited, though the comorbidity-driven pathophysiology of HFpEF makes it a promising subject. In addition, despite the technological advances in multi-omics, knowledge of molecular characteristics of HFpEF remains limited, possibly due to difficulties of biopsy acquisition in HFpEF patients [16] and heterogeneity of HFpEF patients [17].

In this study, we applied a network medicine approach to describe comorbidity patterns in HFpEF and investigate a shared genetic background associated with these patterns. We first demonstrated that comorbidity profiles vary between HFpEF and HFrEF patients and derived distinct comorbidity profiles for each cohort. Then, we built a comorbidity network that contained disease clusters relevant for HF patients. The construction of a multilayer heterogeneous network by integration of prior knowledge resources allowed us to translate the comorbidity profiles into a gene signature for HFpEF. We corroborated this signature in the cardiac transcriptome of a murine HFpEF model. This network medicine approach allowed us to identify distinct comorbidity profiles and novel genetic patterns in HFpEF.

## Methods

### Study population

**Fig. 1:**
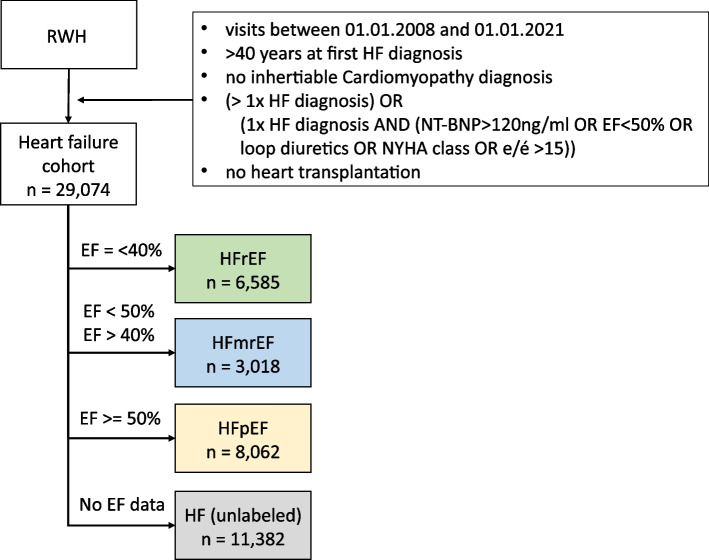
Patient cohort description. Phenotyping algorithm to define HF cohorts. HF patients were selected with hospital visits over a time span of 13 years at the University Hospital Heidelberg. We defined a general HF cohort by selecting patients with either two or more HF relevant ICD-10 codes or one HF relevant ICD-10 code and one additional HF relevant clinical characteristic, yielding 29,047 HF patients. Based on LvEF, we subclassified HF patients to HFrEF, HFmrEF, or HFpEF. RWH Research Data Warehouse, HF heart failure, LvEF left ventricular ejection fraction; e/e’ is the ratio between early mitral inflow velocity and mitral annular early diastolic velocity on echocardiography

The study population was derived from a research data warehouse containing data from patients that visited the Department of Cardiology, Angiology, and Pneumology at Heidelberg University Hospital, Heidelberg, Germany [18]. Heidelberg University Hospital acts as a tertiary care center for the surrounding region, specializing in the treatment of cardiomyopathy. From this data warehouse, we identified patients with HF, visiting between 01.01.2008 and 01.01.2021. The study protocol was approved by the local ethics committee. HF was defined as two or more HF-relevant International Classification of Disease, version 10 (ICD-10) diagnosis codes (I50*, I11.0, I13.0, I13.2, I42.0, I42.5, I42.8, I42.9, I25.5) or at least one HF-relevant diagnosis and at least one of the following criteria: (i) elevated N-terminal pro b-type natriuretic peptide (NTproBNP) (> 120 ng/ml), (ii) recorded New York Heart Association functional class, (iii) echocardiography based E/e’ > 15 ( ratio of early diastolic mitral inflow velocity to early diastolic mitral annulus velocity), (iv) echocardiography or MRI-based left ventricular ejection fraction (LvEF) < 50%, and (v) documented loop diuretic. Patients with HF before age 40, those with a diagnosis of inheritable cardiomyopathy (I42.1-I42.4, I42.6, I42.7), and heart transplant patients (Z94.3) were excluded from the HF cohort. Within the HF cohort, HF subtypes were identified, based on echocardiographic or MRI-based LvEF. Patients with LvEF ≥ 50% were labeled HFpEF, LvEF 40–50% HFmrEF (HF with mid-range ejection fraction), and ≤ 40% HFrEF (Fig. 1). For all patients in the HF cohort, demographics, ICD-10 codes, operational and procedural codes, and targeted clinically relevant measurements were processed (Additional file 1: Fig. S1A, B) [11, 19–38].

### Multiple correspondence analysis (MCA)

Disease profiles of HFpEF, HFrEF, and HFmrEF cohorts were captured as binary variables (0—patient has no record, 1—patient has a record of disease) of 569 phenome-wide association scan codes (PheCodes) (Additional file 1: Supplementary Methods). In this feature space (569 comorbidities × 17,665 HF patients), we performed MCA (R-package *FactoMiner* [39]). Each MCA dimension was then tested for association with clinical covariates with linear regression models (e.g., MCA-dimension 1 ~ age). For each covariate, we then summed the variance associated to all significantly associated dimensions (*p*-value < 0.05) as an estimate for the total associated variance.

### Patient classifier

We trained random forest and regularized logistic regression (elastic net) models to predict HFpEF and HFrEF cohort labels on 569 PheCodes. The highest achieved mean area under the receiver operating characteristic (AUROC) in hyperparameter tuning was reported as an estimate for the model test error (Additional file 1: Supplementary Methods).

To derive the comorbidity profiles for HFpEF and HFrEF, we performed a forward selection with an L1-regularized logistic regression model of the 196 non-zero features from the elastic net model. Models were trained in R with R packages *tidymodels* using model engines from *glmnet* [40] and *ranger* [41]*.*

### HF comorbidity network (HFnet) construction

In disease comorbidity networks, nodes represent diseases while edges represent statistical association of co-occurrence, resulting in the graphical depiction of comorbidities as diseases that are statistically dependent. In detail, we selected edges using Fisher’s exact test for estimating statistical dependence and its Benjamini–Hochberg (BH) corrected *p*-value (< 0.0001) to discard non-significant disease pairs and keep a more sparse network structure. To determine strength of association, we calculated *ɸ* correlation, which can be interpreted as a Pearson correlation for binary variables. We selected all edges with positive correlation. To account for bias in *ɸ* correlations, we scaled the values by dividing by mean correlation values for every disease and assigned these values as edge weights [42].

Network node characteristics, such as betweenness, closeness, and degree centrality, and transitivity were calculated with the *igraph* R package. To calculate metrics based on graph distance, we replaced weights for edge *i* (*W*_*i*_) with a new edge score *S*_*i*_: $S_i= \mathrm{max}(W)-W_i$. The constructed network was then compared to other comorbidity networks (Additional file 1: Supplementary Methods).

### HF heterogeneous network (HFhetnet) construction

#### Disease-gene association

We used disease–gene associations provided by DisGeNet v7.0 [43, 44] and mapped the ICD-10 codes in DisGeNet to PheCodes (Additional file 1: Supplementary Methods). To ensure that the most frequent diseases in our cohort were mapped, we selected the most frequent 3-digit ICD-10 codes that were not mapped to DisGeNet and performed manual annotation via Unified Medical Language System (UMLS) IDs for 23 disease entities (Additional file 2: Supp. Table 1), e.g., PheCode *427.2 (*atrial fibrillation) was manually mapped to the UMLS ID *C000423.* We only included disease–gene associations with a DisGeNet confidence score > 0.29. This cut-off was chosen, such that either one curated source or multiple experimental sources were necessary for disease–gene associations. Details on DisGeNET score calculation can be found at https://www.disgenet.org/dbinfo.

#### Gene–gene association

To consider multiple layers of gene organization, we constructed a multilayer gene network from different sources.

Omnipath [45, 46] is a meta resource of a multitude of biological knowledge databases, and we curated a network by connecting two genes if a resource provides a co-membership for a signaling pathway. We used the number of resources that reported a relationship as an estimate for the confidence in the relationship, which we introduced as edge weights in the Omnipath layer.

The protein–protein interaction (PPI) network was constructed based on the union of publicly available data from experimental and literature curated data (HuRi-union [47]) [48].

Gene Ontology (GO) gene networks have been constructed before, and we used the GO networks constructed by [49].

Each gene network was reduced to remove loops and multiple edges. To filter for genes relevant in cardiac tissue, gene networks were subset to genes expressed in the human heart on RNA or protein level. For protein expression, we used proteomic data [50, 51], where we selected all peptides that were detected in the human heart and used the leading gene associated with each peptide. For gene expression, we selected genes that were detected in the heart tissue in the Genotype-Tissue Expression (GTEx) Project v8 with a *transcript per million* value > 1 [52]. We chose this threshold to discard non-expressed genes but include lowly expressed genes. To ensure that gene programs only active in diseased hearts were also captured, we also included genes that were captured in a meta-analysis of HF transcriptomes [32, 33].

### Disease–gene prediction and prioritization

To predict genes from diseases within the HFhetnet, we relied on a network propagation algorithm developed for multilayer networks (random walk with restart on multiplex heterogeneous networks; RWR-MH [53]). This algorithm is an extension of the random walk algorithm that tries to find a stationary distribution of probabilities that a node is visited when a random walk on the network is initiated in a set of seed nodes.

We assessed the performance of the link prediction task within the HFhetnet. For a given disease that was present in the HFhetnet and directly linked to two or more genes, we attempted to predict those genes after removing the direct links from the HFhetnet and running RWR-MH with the disease as seed node. The position of the target genes in the resulting probability ranking was then assessed with multiple metrics to estimate success of disease gene recovery (Additional file 1: Supplementary Methods).

For HFpEF and HFrEF gene prediction, we applied the RWR-MH, using the comorbidity profiles from the patient classifier as the seed nodes in the HFhetnet. This yielded two vectors of RW probabilities for each comorbidity profile. The top 500 genes yielded non-zero probability values for each profile. To select gene candidates that were differently ranked, we calculated a prioritization score for HFpEF and HFrEF. For this, we calculated $G_i= {P_i}*|\Delta R_i|$.

*G* is the gene prioritization score, *P* is the RW-based probability, Δ*R* is the rank difference between HFpEF and HFrEF rankings for gene *i*.

### Transcriptome analysis

We filtered lowly expressed genes and normalized samples using the Trimmed mean of M-values (*edgeR* [54]) and subsequent variance-stabilizing transformation (*limma voom*) and performed differential expression analysis (*limma *[55]. We performed principal component analysis and Gene Ontology enrichment with the *enrichr *[56] R package. For the overrepresentation analysis, we ranked genes by t-statistic and performed gene set enrichment analysis (*fgsea* R package [57]) of the top predicted HFpEF and HFrEF genes using different cut-offs.

## Results

### The study population

The study population consisted of 29,047 patients with HF (Fig. 1). Within this cohort, we identified three sub cohorts, HFpEF (8062 patients), HFrEF (6585 patients), and HFmrEF (3018 patients) based on LvEF. LvEF was not recorded in 11,382 HF patients, preventing subcohort labeling (i.e., unlabelled HF cohort). HFpEF patients were more often female compared to HFrEF patients (35 vs 25%, *p* < 0.01) (Table 1). However, we did not observe a significant difference in body mass index (median [IQR] = 26.8 [24.2, 30.0] vs 26.5 [24.1, 30.1] for HFpEF vs HFrEF, *p* = 0.9) or age (median [IQR] = 70 [61, 88] for HFpEF vs 70 [60, 70] for HFrEF, *p* = 0.5). When phenotypic data were available, cholesterol, LDL, HDL, and blood pressure values were higher in HFpEF patients compared to HFrEF, while NT-proBNP values were higher in HFrEF patients. Comorbidity burden measured by Elixhauser index was slightly lower in HFpEF than HFrEF patients, as previously reported [58]. HFpEF patients were intubated (8.5% vs 15%, *p* < 0.001) or received an implantable cardioverter-defibrillator (16% vs 26%, *p* < 0.001) less frequently than HFrEF patients, suggesting that the HFrEF cohort be a later stage of HF.

**Table 1:** Clinical characteristics of HFrEF, HFmrEF, and HFpEF cohorts. Descriptive statistics of HFrEF, HFmrEF and HFpEF cohorts. F female, m male, BMI body mass index, BP blood pressure, LDL low-density lipoprotein, HDL high-density lipoprotein, ICD implantable cardioverter defibrillator, PCI percutaneous coronary intervention, NT-BNP N-terminal pro b-type natriuretic peptide. All numerical values are median (IQR), Elixhauser index is mean (SD)

			HF subtypes				
Variable	*N*	Overall	HFpEF	HFmrEF	HFrEF	*p*-value^*^	*p*-value^+^
		(*N* = 17,665)	(*N* = 8062)	(*N* = 3018)	(*N* = 6585)		
Sex	17,617					< 0.001	< 0.001
Female		5247 (30%)	2822 (35%)	790 (26%)	1635 (25%)		
Male		12,370 (70%)	5228 (65%)	2218 (74%)	4924 (75%)		
Age (years)	17,665	70 (60, 78)	70 (61, 78)	70 (59, 77)	70 (60, 78)	0.093	0.5
BMI	9132	26.8 (24.2, 30.0)	26.8 (24.2, 30.0)	26.9 (24.2, 29,9)	26.5 (24.1, 30.1)	0.08	0.9
Systolic BP (mmHg)	5146	148 (134, 160)	150 (139, 164)	148 (135, 160)	140 (127, 154)	< 0.001	< 0.001
Diastolic BP (mmHg)	5146	84 (76, 92)	85 (78, 93)	84 (76, 93)	82 (73, 90)	< 0.001	< 0.001
LDL (mg/dL)	12,270	87 (69, 110)	88 (69, 110)	91 (72, 113)	84 (67, 106)	< 0.001	< 0.001
HDL (mg/dL)	12,368	44 (36, 54)	46 (38, 56)	43 (36, 53)	40 (34, 50)	< 0.001	< 0.001
Triglycerides (mg/dL)	13,859	112 (85, 153)	112 (85, 151)	112 (85, 156)	113 (85, 156)	0.11	0.006
Cholesterol (mg/dL)	13,577	160 (135, 188)	163 (138, 190)	164 (140, 192)	153 (129, 183)	< 0.001	< 0.001
N PheCodes	17,665	13 (8, 21)	13 (9,21)	12 (7, 19)	14 (9, 22)	< 0.001	0.088
Elixhauser Index	17,665	5.39 (2.72)	5.36 (2.68)	5.09 (2.70)	5.56 (2.76)	< 0.001	< 0.001
Intubated	17,665	1766 (10.0%)	552 (6.8)	257 (8.5%)	957 (15%)	< 0.001	< 0.001
ICD Implantation	17,665	3213 (18%)	1007 (12%)	468 (16%)	1738 (26%)	< 0.001	< 0.001
PCI	17,665	9116 (52%)	4267 (53%)	1554 (51%)	3295 (50%)	0.002	< 0.001
log (NT-BNP)	6169	2.99 (2.45, 3.53)	3.07 (2.53, 3.55)	3.07 (2.53, 3.55)	3.45 (2.96, 3.88)	< 0.001	< 0.001

All continuous values displayed as median (IQR) except for Elixhauser index which is mean (SD). All dichotomous values displayed as *N* (%)

^*^Kruskal–Wallis *p*-value across all subtypes

^+^Wilcoxon–rank sum or chi-squared *p*-value for HFpEF vs HFrEF

### High variation in comorbidity profiles is associated with HFpEF/HFrEF subtype

**Fig. 2:**
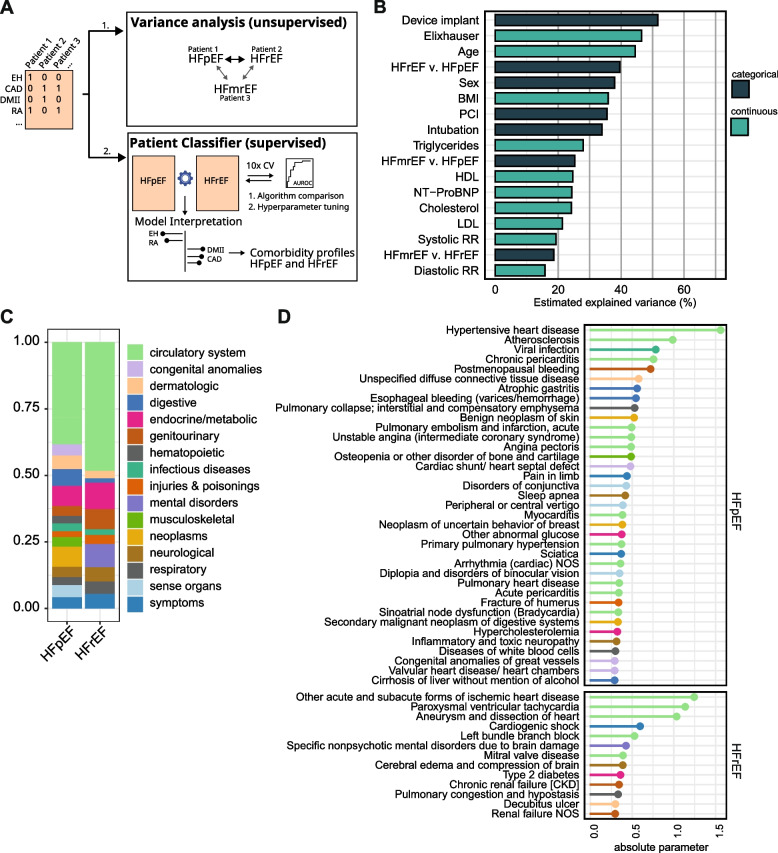
Comparison of comorbidity profiles in heart failure subtypes. **A** Scheme of analysis. EH essential hypertension, CAD coronary artery disease, DMII diabetes mellitus type II, RA rheumatoid arthritis. **B** Multiple correspondence analysis of comorbidity profiles of HFpEF and HFrEF cohort. MCA dimensions were tested for association with clinical covariates and summed up to estimate total explained variance. **C** Proportions of the sum of parameter estimates of top 100 comorbidities of the patient classifier model, colored by disease categories. **D** Top 50 comorbidities of the patient classifier. The parameters are the absolute fitted values of the coefficients in the elastic net model for each comorbidity of the patient classifier separated by association to HFpEF (top) or HFrEF features (bottom). Colors indicate disease category using the same color legend as in **B**

We expected differences in the composition of comorbidity profiles between HF subtype cohorts. To quantify this variance, we applied MCA and estimated the variance associated with sub-cohort labels and clinical features (Fig. 2A). Device implantation was the feature most strongly associated with variance in comorbidity profiles (Fig. 2B). When comparing HF subtypes, HFpEF and HFrEF cohort labels were associated with a high degree of explained variation (39.5%). HFmrEF patients seemed to be in an intermediate state, as they displayed lower variance when compared to HFpEF (25.2%) and HFrEF (18.6%). Sex and age were each associated with high variance (37.9% and 44.4%, respectively) as expected. In summary, this analysis approach identified a pronounced contrast between comorbidities in HFpEF and HFrEF patients.

### Distinctive comorbidity profiles derived for HFpEF and HFrEF

Next, to explain and interpret the variance between HFpEF and HFrEF, we derived distinct comorbidity profiles for both cohorts. For this purpose, we fit random forest and elastic net classifier models with the 569 comorbidities as predictors to distinguish between HFpEF and HFrEF (Fig. 2A, Additional file 1: Fig. S2A,B). The highest achieved AUROCs were 0.778 for the random forest and 0.777 for the elastic net model, indicating that the random forest’s ability to model more complex interactions between comorbidities did not improve classifier performance substantially. The most important features were shared in both models (Additional file 1: Fig. S2C).

Next, because elastic net parameter estimates can provide both magnitude and direction, we selected the elastic net model to assign HFpEF and HFrEF a distinctive set of comorbidities. To select the most discriminant comorbidities, we performed forward selection. We found that the model with 100 comorbidities yielded a cross-validated AUROC of 0.780 (Additional file 1: Fig. S2D); 71 and 29 comorbidities from this model were assigned to HFpEF or HFrEF, respectively, which we will refer to as their comorbidity profiles.

These comorbidity profiles might be influenced by age, sex, time of visit, or time relative to HF diagnosis. We therefore investigated whether these factors influence the assignment of these 100 comorbidities to HF subtype by fitting a series of logistic regression models in different data subsets (Additional file 1: Supplementary Methods). We found that the derived comorbidity profiles of HFpEF and HFrEF yielded mostly consistent patterns independent of these factors (Additional file 1: Fig. S4).

The HFpEF profile (15 disease categories) was more diverse than the HFrEF profile (10 disease categories) and included comorbidities from the digestive disease, hematopoietic and neoplastic disease categories (Fig. 2C). Cardiovascular disease was the most important category in both profiles, accounting for 48.2% of the sum of parameter estimates in HFrEF and 38.3% in HFpEF. In HFpEF, important comorbidities included hypertensive and pulmonary heart disease, essential hypertension, inflammatory cardiac conditions (pericarditis, myocarditis), sleep apnea, osteopenia, neoplasms (multiple myeloma, breast cancer, metastasis in digestive systems), and rheumatoid disorders. The HFrEF comorbidity profile was characterized among others by myocardial infarction, ischemic heart disease, tobacco abuse, mitral valve disease, coma and cardiogenic shock, neurological disorders (vascular dementia, cerebral edema), chronic kidney disease, and diabetes type II (Fig. 2D, Additional file 1: Fig. S3).

In conclusion, the observed variation in comorbidity profiles between HFpEF and HFrEF was analyzed by interpreting patient classifiers. The derived features captured known subtype associations such as typical etiologies of HF including hypertensive heart disease (with HFpEF) and ischemic heart disease (with HFrEF) but also more novel and understudied comorbidities associated with HFpEF such as breast cancer or rheumatoid arthritis with HFpEF.

### The HF comorbidity network (HFnet) captures HF specific disease relationships

**Fig. 3:**
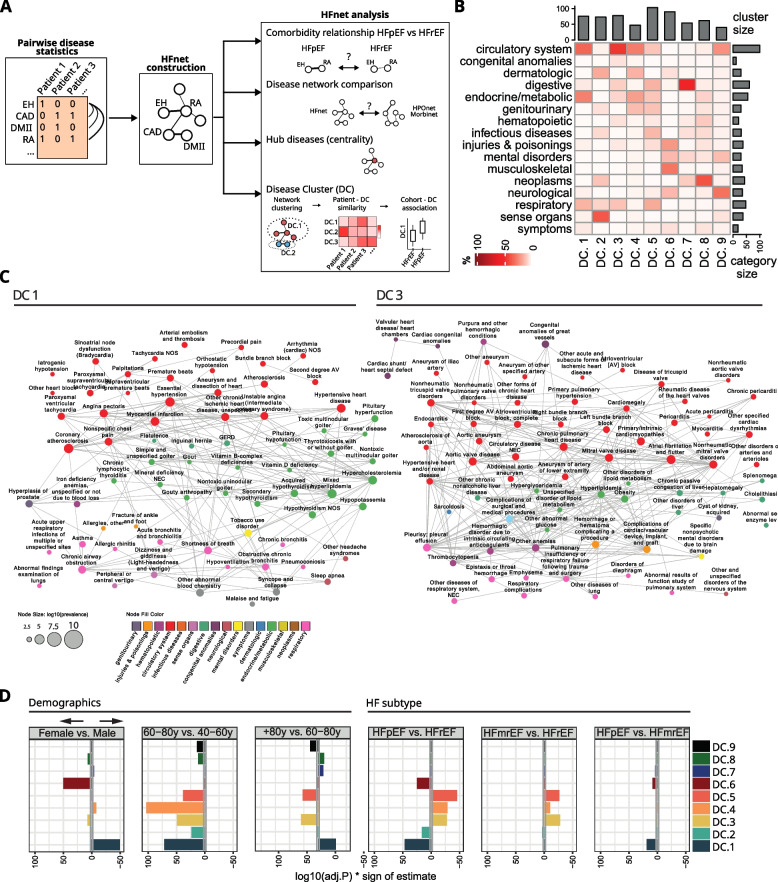
The heart failure comorbidity network (HFnet). **A** Scheme of comorbidity network analysis. EH essential hypertension, CAD coronary artery disease, DMII diabetes mellitus type II, RA rheumatoid arthritis. **B** Disease category composition of disease clusters (DCs) in the HFnet. Number of nodes per cluster in top barplot and number of diseases per category in side barplot. **C** Subgraphs of the HFnet visualized (left DC1, right DC3). Node size relates to prevalence, edge width to scaled phi-correlation, node color to disease category. Only edges with highest weights were plotted for visibility. **D** Comparison of patient cohorts based on DC similarity. Jaccard indices were calculated between each patient and each DC, then unpaired two-sided Wilcoxon rank test was applied to compare different patient cohorts. The log transformed *p*-value was multiplied by the sign of the test estimate for visualization purposes such that positive values indicate higher cluster similarity with the first cohort of the contrast label. Patient cohorts were selected by age stratification, sex, and HF subtype

To analyze patterns of disease co-occurrence in the HF-patient cohort, we constructed a comorbidity network as previously described [11, 12, 59–61]). This network was built by calculating pairwise disease correlations for the general HF-patient cohort (Fig. 3A) (Additional file 1: Fig. S6A,B). The resulting significant disease–disease relationships were assembled to form an undirected and weighted HF comorbidity network (HFnet) consisting of 569 nodes and 19,347 edges (Additional file 1: Fig. S6C), with edge weights defined by a statistical dependency of co-occurrence for each disease pair.

While certain comorbidities were distinctive for HFpEF or HFrEF, it was unclear whether the disease relationships that built the HFnet also depended on the HF subtype (Fig. 3A). When comparing odds ratios for each disease pair from both cohorts, we found a high concordance (Additional file 1: Fig. S5A,B). Only 33 disease pairs had significantly different odds ratios between HFpEF and HFrEF (Breslow–Dayes test with BH correction *p* < 0.01) (Additional file 1: Fig. S5C), suggesting that in the vast majority of cases, the co-occurrence of two diseases did not depend on whether it was assessed in HFpEF or HFrEF patients.

Whether the HFnet constituted a unique wiring of diseases or predominantly captured generalizable disease relationships was unclear. To investigate this, we analyzed and compared two additional disease networks: a human phenotype ontology network (HPOnet), where two diseases are connected if they are phenotypically similar, and Morbinet [11], another comorbidity network from a large patient cohort but without a cohort defining disease (Additional file 1: Supplementary Methods).

Jaccard index-based edge similarity of HFnet and Morbinet was 0.18 and of HFnet and HPOnet was 0.12 (Additional file 1: Fig. S7A,B,C). We then calculated network similarities with the DeltaCon algorithm to capture conserved node affinities between networks [28]. HFnet and Morbinet displayed again a higher similarity (0.46) than HFnet and HPOnet (0.39) (Additional file 1: Fig. S7D). This suggested that comorbidity correlation was not completely redundant with phenotype similarity. The differences between Morbinet and HFnet indicated that many disease relationships in the HFnet could be specific for HF patients.

Finally, we analyzed the centrality of diseases. Diseases which were most frequently reported could be considered the network hubs, as indicated by their high node degree and their closeness and betweenness centrality scores (Additional file 1: Fig. S8A). Our network captured well-known HF comorbidities [41, 42], like chronic kidney disease, which by multiple metrics was the main HFnet hub (Additional file 1: Fig. S8B, Additional file 3: Supp. Table 2). We found that closeness and degree centrality were both significantly associated with the disease category (Additional file 1: Fig. S8C) (Kruskal–Wallis *p* < 0.01). Infectious and hematopoietic diseases had the highest median centrality scores (betweenness, closeness, and degree), indicating that patients with diseases from these categories were typically suffering from many comorbidities. Diseases affecting the circulatory system had the highest prevalence as was expected with a HF centered cohort (Additional file 1: Fig. S8C).

In summary, we found that comorbid relationships were mostly independent of the HF subtype. However, when comparing with other disease networks, many comorbid relationships were specific to the HFnet. This indicated that the constructed HFnet captured disease patterns relevant for HF patients, which only in part can be generalized to other cohorts.

### The HFnet contains 9 disease clusters that represent important comorbidity groups in HF

Network communities represent densely connected subgraphs and can be helpful to summarize network topology. Network clustering algorithms identified nine disease clusters (DCs) (Additional file 1: Supplementary Methods, Fig. S9A-F). DCs were partially grouped by disease categories (Fig. 3B, Additional file 4: Supp. Table 3) and we labeled DCs by manually reviewing disease composition (Table 2). For instance, DC1 and DC3 contained the majority of cardiovascular diseases. While DC1 contained cardiovascular diseases with vascular etiology (EH, CAD, MI) and included metabolic and endocrine diseases, DC3 contained valve disorders and arrhythmias (Fig. 3C).

**Table 2:** Overview of disease clusters. Manual labeling of disease clusters (DC) by characterizing most central and prevalent diseases in each cluster. EH essential hypertension, MI myocardial infarction, COPD chronic obstructive pulmonary disease, CAD coronary artery disease, DM II diabetes mellitus type II, CKD chronic kidney disease, RA rheumatoid arthritis

DC	Label	Important nodes
DC1	Cardiac/endocrine/respiratory diseases	EH, MI, COPD, hyperlipidemia, hypothyroidism, CAD
DC2	Sensory/ophthalmologic/skin disease	Cataract, macular degeneration, melanomas of skin
DC3	Cardiovascular disease with heart focus	Valve disease, congenital anomalies, arrhythmias
DC4	Vascular/renal/diabetic diseases	DM II, CKD, atherosclerosis
DC5	Critical illness/complications	Infectious disease, organ failures
DC6	Rheumatoid/osteological/psychiatric diseases	Osteoporosis, osteopenia, RA, depression
DC7	Gastroenterological diseases	Gastritis, diverticulitis, cirrhosis
DC8	Neoplastic/hematopoietic diseases	Breast cancer, aplastic anemia, lymphomas
DC9	Neurological/vascular neurological diseases	Stroke, dementias, epilepsy

We hypothesized that DCs represent facets of the subcohort specific HF comorbidity spectrum, and we therefore tested whether DCs capture demographic or HF subtype-related characteristics. We quantified the similarity of an individual patient's comorbidity profile with each DC by calculating Jaccard indices and tested for differences between patient cohorts (Fig. 3D). In age-stratified analyses, we found that all DCs, except DC7, were more similar to 60–80-year-old (*n* = 16,54) compared to 40–59-year-old patients (*n* = 5973) comorbidity profiles. This could indicate a general increase of comorbidity burden with age or that with age come increasingly consistent comorbidity profiles between individuals. The 80 + cohort (*n* = 6,527) had less similarity with DC1 and significantly more similarity with DC3, DC5, and DC9 profiles compared to 40–60-year-old patients. When comparing female and male patients, we found that DC6 and DC2 yielded the highest similarity differences, respectively. Comparing HFpEF with HFrEF patients, we found that DC1, DC2, DC6, and DC8 were more similar to HFpEF patients, while DC3, DC4, and DC5 were suggested to be similar to HFrEF patients. As DC1 and DC6 also captured sex-related comorbidity differences, we investigated further, whether DC6 diseases were more prevalent in HFpEF independent of sex. For this, we fit logistic regression models for each disease predicting HFpEF/HFrEF while controlling for sex (Additional file 1: Fig. S9G). Again, DC1, DC2, and DC6 contained more diseases prevalent in HFpEF while DC3, DC4, and DC5 diseases were more prevalent in HFrEF. In addition, this analysis also suggested that many diseases in DC7 and DC8 too were distinctive for HFpEF.

We further compared the comorbidity profiles from the patient classifier by mapping them to DCs which yielded a qualitatively similar DC to HF subtype association (Additional file 1: Fig. S9H). No DC was positively associated with HFmrEF. Instead, HFmrEF patients were less similar to DC1 and DC6 than HFpEF patients and less similar to DC3, DC4, and DC5 than HFrEF patients.

In general, we found that aggregating comorbidity profiles (569 dimensions) to DC similarity (9 dimensions) allowed us to capture differences among patient cohorts in regard to sex, age, and HF subtype in meaningful disease groups.

### Building the HF heterogeneous network (HFhetnet)

Biomedical research has yielded significant knowledge of disease gene associations, which can be harnessed to extrapolate novel disease gene relationships. HFpEF is a comorbidity-driven syndrome and we hypothesized that the identified HFpEF comorbidity profile could be translated to a genetic profile consisting of recurrent genetic associations to these comorbidities. In this part of our study, we first integrated multiple biomedical databases to construct a cardiac specific multilayer disease and gene network. We then estimated the success of this network to recover known disease–gene associations and, finally, used the HFpEF comorbidity profile to identify the most commonly associated genes.

**Fig. 4:**
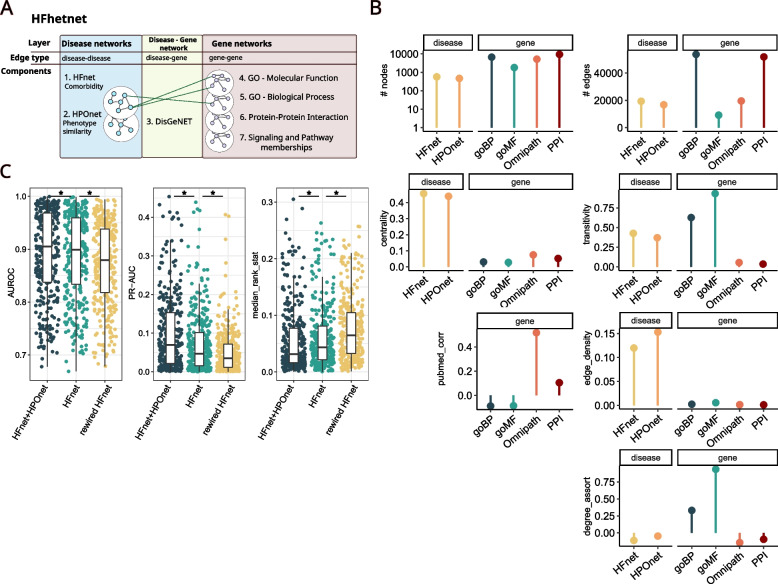
HFhetnet characterization. **A** Schematic overview of HFhetnet and its different layers built by including seven independent data sources. **B** Characterization of network layers by size (number of nodes and edges), edge density (percentage of possible edges), degree centrality, global transitivity (average probability of the neighbors of a node being connected), degree assortativity (preference of nodes to connect with nodes of similar degree), and literature bias (i.e., gene degree/PubMed score correlation). **C** Leave one out cross-validation results for all diseases with two or more DisGeNET links. We compared the performance of gene set recovery with different versions of the HFhetnet by modifying only the disease network. We compared HFnet + HPOnet (i.e., the original HFhetnet), only the HFnet (without HPOnet), and a rewired HFnet. Outliers are not plotted for visualization purposes. Paired, two-sided Wilcoxon test, *p < 0.001. AUC-PR area under the precision/recall curve, AUROC area under the receiver operator curve. GO Gene Ontology, HPO human phenotype ontology

To construct a gene network that reflected different hierarchies of gene function (i.e., pathway memberships, PPI, and ontological similarity), we integrated multiple databases and represented gene–gene relationships as networks (“Methods”) (Fig. 4A). To focus on genes relevant in cardiac tissue, we subset the resulting gene networks to protein coding genes expressed in the heart (Additional file 1: Fig. S10A). Next, we used DisGeNET, a resource containing disease–gene associations, to connect the HFnet with the gene network. We connected 400 diseases of the HFnet with a total of 4044 genes via 20,170 edges. As the HPOnet constructed earlier had a small intersection with the HFnet and captured a different type of disease relationship, it was included as an additional disease layer in our network.

The presented HFhetnet is an assembly of the data-driven comorbidity relationships (HFnet) and six biomedical databases resulting in a total of 13,572 nodes and 181,529 edges (Additional file 5: Supp. Table 4). Its main structure is set up by two biological networks (disease layer and gene layer) that each consist of two or four network layers, respectively (Fig. 4A). The two disease networks were the smallest when comparing node numbers (Fig. 4B). However, edge density was much higher resulting in centralization of these networks compared to the gene layers. Within gene layers, the ontological layers displayed the highest transitivity, as well as tendency to connect to hub genes (degree assortativity). To assess research bias in the gene networks, we calculated Pearson correlation between the number of abstracts in PubMed mentioning a gene and the gene’s network degree per layer and found that only the pathway layer (Omnipath) displayed significant correlation (*p*-value < 0.05). This is related to a biomedical research bias towards the investigation of a small number of genes [62]. Thus, the integration of experimental and ontological data can ameliorate the centrality of overstudied genes.

In summary, we constructed the HFhetnet by integrating various prior knowledge resources to incorporate genetic information. The different network layers of the HFhetnet captured unique node relationships and displayed particular network topologies.

### Estimating the success of disease–gene prediction within the HFhetnet

To estimate the potential of the HFhetnet to predict disease–gene relationships, we estimated the success of predicting known disease genes. The rationale behind this approach is the guilt-by-association principle that assumes that functionally related genes are also associated in the network context. Extending this notion to heterogeneous networks, this principle can be interpreted as a disease being associated with relevant disease genes through its position in the network. To quantify this property, we applied a leave-one-out validation design to assess whether known disease genes can be recovered after removing the direct edges that connected them to a disease. After edge removal, the gene recovery was performed by applying the RWR-MH algorithm which considers each network layer and its topology ( Additional file 1: Supplementary Methods).

We performed this analysis by comparing the impact of three variations of the disease layer: (i) HFnet + HPOnet (original HFhetnet), (ii) only HFnet, and (iii) a rewired HFnet. Gene prediction worked best in the original HFhetnet (median AUROC 0.91, median AUC-PR 0.07, and median rank ratio 0.03) (Fig. 4C). This performance dropped for every metric when removing the HPO layer or when rewiring the HFnet (paired, two-sided Wilcoxon’s rank sum test *p* < e − 10). The rewired HFnet still performed better than random, which might be explained by (i) high edge density in the HFnet and (ii) the large size of the unaltered gene–gene and disease–gene network in comparison to the smaller HFnet.

Prediction success correlated weakly but significantly with gene set size (Additional file 1: Fig. S10C). In addition, neither disease prevalence nor DisGeNET confidence scores were significantly correlated with prediction success, suggesting that frequent diseases could not be predicted better than less frequent diseases. Prediction performance depended on disease category (Additional file 1: Fig. S10D) (Kruskal–Wallis test *p*-value < 0.01 for all metrics) with respiratory, neurological, genitourinary, and cardiovascular diseases performing best.

In summary, we found that within the HFhetnet, the disease genes remained close via the disease’s connection through its comorbidities or phenotypically similar neighbors. Thus, we concluded that HFnet and its extension, HFhetnet, captured meaningful disease–disease, disease–gene, and gene–gene relationships, which can be exploited for predicting a disease’s genetic profile through its comorbidities.

### Predicting genes associated with comorbidity profiles of HFpEF and HFrEF

In the first part of our study, we found that HFpEF and HFrEF patients were distinguishable based on their comorbidity profiles. We then demonstrated that diseases within the HFhetnet were located in network proximity to their respective disease–genes. Leveraging both insights, we hypothesized that genes located close to the HFpEF and HFrEF comorbidity profiles could yield novel candidates for the respective HF subtype. In this section, we applied the RWR-MH algorithm with the HFpEF and HFrEF comorbidity profiles as seed nodes resulting in gene ranking based on network proximity (Additional file 1: Fig. S11A-C).

**Fig. 5:**
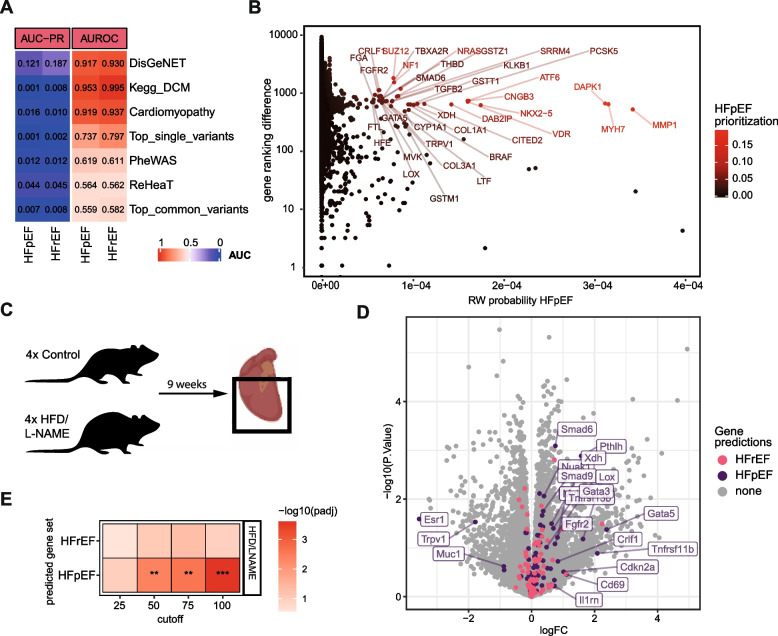
HFpEF gene prediction. **A** AUROC and AUC-PR for different HF-related gene sets in random walk probability vectors based on HFpEF and HFrEF comorbidity profiles. Prior knowledge gene sets are DisGeNET, Kegg pathway for dilated cardiomyopathy (DCM), cardiomyopathy (literature curated). Data-based gene sets are PheWAS, ReHeaT, and GWAS variants. **B** Prioritizing genes for HFpEF that are close to HFpEF comorbidity profiles in the HFhetnet and also display high ranking differences when compared to gene predictions based on HFrEF comorbidity profiles. **C** Scheme of experimental design for murine model of HFpEF by HFD/L-NAME diet. Cardiac ventricles were harvested after 9 weeks and bulk transcriptomics were collected. **D** Volcano plot displaying gene expression regulation in the murine HFpEF model compared to control. Labeled genes display HFpEF predicted genes from human comorbidity profiles. **E** Predicted HF genes from comorbidity analysis were enriched in gene-level t-statistics of murine differentially expression analysis comparing disease with control. Gene set enrichment *p*-value. ***p < 0.001. **p < 0.01

To assess whether the resulting gene rankings recapitulated known HF genes, we curated a set of HF-related genes from various prior knowledge sources and independent datasets (Additional file 1: Supplementary Methods), which had only little intersection (Additional file 1: Fig. S11D). We found that prior knowledge gene sets were well recovered within the HFpEF and HFrEF gene rankings (Fig. 5A, Additional file 1: Fig. S11E). Gene sets that were retrieved from experimental data (gene expression, PheWAS, GWAS) performed worse in the predictions. Next, we compared these prediction results with random comorbidity profiles and found that the HFrEF profile associated with Kegg’s dilated cardiomyopathy (DCM) (z-score AUROC 1.77; z-score PR-AUC 6.7) and DisGeNETs HF genes (z-score AUROC 1.76; z-score PR-AUC 2.46) (Additional file 1: Fig. S11F). This indicated that the HFrEF comorbidity profile which was more cardiac centered was closer to prior knowledge of HF genes within the HFhetnet. In general, well-known genes relevant for HF were recovered for both, HFpEF and HFrEF comorbidity profiles including NPPA, NPPB, TNFa, NOS2, NOS3, CCL2, IL1B, LMNA, and TTN (Additional file 1: Fig. S11D).

To emphasize genes that might be HFpEF specific, we prioritized genes that were close to the HFpEF but not to the HFrEF comorbidity profile within the HFhetnet (Fig. 5B, Additional file 6: Supp. Table 5, Additional file 1: Supplementary Methods). We found that MMP1, MHY7, and DAPK1 received the highest scores and other candidates included genes functionally involved in fibrosis (e.g., LOX), metabolism (MVK), transcriptional regulation (ATF6), coagulation (THBD), and oxidative stress (NOS1, XDH) (Table 3).

**Table 3:** Potential HFpEF candidates based on network proximity to comorbidity profiles. Genes are sorted by functional groups. Rank indicates prediction rank for HFpEF. *TF* transcription factor, *CM* cardiomyopathy

Rank	Gene symbol	Gene name	Functional group	Gene function	Role in HF (exemplary or putative)	References
17	PCSK5	Proprotein convertase subtilisin/kexin type 5	Cell differentiation	Mediates post translational endoproteolytic processing	Cleaves GDF1, heart development	[63, 64]
9	NKX2-5	NK2 Homeobox 5	Cell differentiation	TF expressed in precursor cardiac cells, involved in cardiac development	Heart development, activated in HF involved in hypertrophy	[65, 66]
31	GATA5	GATA binding protein 5	Cell differentiation	TF, involved in embryonic development of the heart	Possible role in cardiac hypertrophy, linked to dilated CM	[67]
42	GATA3	GATA binding protein 3	Cell differentiation	TF, involved in embryonic development of the heart and immune cell differentiation	Role in T-cell recruitment	[68, 69]
67	JAG1	Jagged canonical Notch ligand 1	Cell differentiation	Interacts with four receptors in the mammalian Notch signaling pathway	Possible protective role against PAH and diabetic CM, involved in regenerative capacity of the heart	[70–72]
62	NOTCH1	Notch receptor 1	Cell differentiation	Regulates interactions between physically adjacent cells through binding of Notch family receptors to their cognate ligands	Possible protective role against PAH and diabetic CM, involved in regenerative capacity of the heart	[70–72]
13	CITED2	Cbp/p300 interacting transactivator with Glu/Asp rich carboxy-terminal domain 2	Cell differentiation	TF that controls pluripotency among others	Cardiomyocyte pluripotency	[73]
23	KLKB1	Kallikrein B1	Coagulation	Factor XII activation	Interacts with LDL, possible HF biomarker	[74–76]
27	TBXA2R	Thromboxane A2 receptor	Coagulation	G protein-coupled thromboxane A2 receptor that induces platelet aggregation and regulate hemostasis	Involved in endothelial homeostasis, angiogenesis	[77–79]
34	THBD	Thrombomodulin	Coagulation	Endothelial-specific type I membrane receptor that binds thrombin	Angiogenesis and anticoagulant, possible HF biomarker	[80, 81]
11	DAB2IP	DAB2 interacting protein	Endothelial dysfunction	Ras GTPase-activating protein (GAP)	Associated with endothelial dysfunction in atherosclerosis	[82, 83]
77	ESR1	Estrogen receptor1	Endothelial dysfunction	Estrogen receptor and ligand-activated TF	Regulates NOS activity among many other effects	[84]
7	ATF6	Activating transcription factor 6	ER-stress	Unfolded protein response	Activates XBP1 which modulates ER-stress	[85–87]
3	MMP1	Matrix metallopeptidase 1	Fibrosis	Proteases involved in degrading of ECM	involved in cardiac fibrosis	[88]
25	FGFR2	Fibroblast growth factor receptor 2	Fibrosis	Receptor tyrosine kinases that promote mitogenic signal mediators that induce cell proliferation and survival	Involved in cardiac fibrosis	[89]
33	LOX	Lysyl oxidase	Fibrosis	Cross-linking of collagens	Possible role in myocardial stiffness	[90]
2	MYH7	Myosin heavy chain 7	Hypertrophy	Part of the thick filament in cardiac muscle, involved in contraction	Cardiac hypertrophy marker	[91, 92]
4	SUZ12	SUZ12 polycomb repressive complex 2 subunit	Hypertrophy	Core PRC2 (polycomb repressive complex 2) protein	Mediates long non-coding RNA Ahit induced cardiac hypertrophy	[93]
65	FSTL1	Follistatin like 1	Hypertrophy	Extracellular glycoprotein	Protective against hypertrophy	[94, 95]
79	RGS5	Regulator of G protein signaling 5	Hypertrophy	RGS proteins are involved in the regulation of heterotrimeric G proteins by acting as GTPase activators	Protects from cardiac hypertrophy and fibrosis	[96, 97]
89	CD14	Cluster of differentiation 14	Inflammation	Membrane glycoprotein primarily expressed by myeloid cells that plays a key role in inflammation	Monocytes/macrophages marker in immune disease	[98]
47	SHARPIN	SHANK associated RH domain interactor	Inflammation	Displacing talin from the integrin cytoplasmic domain	Involved in integrin Inactivation and NF-κB Signaling	[99, 100]
90	CD209	Cluster of differentiation 209	Inflammation	Expressed by macrophages and dendritic cells	Involved in rheumatoid arthritis	[101]
36	MVK	Mevalonate kinase	Metabolism	Cholesterol metabolism	Fibrotic effects, Rho family small GTPase activity modulation	[102, 103]
1	DAPK1	Death associated protein kinase 1	Oxidative stress	Calcium/calmodulin-dependent serine/threonine kinase	Protection from oxidative stress in MI	[101]
18	XDH	Xanthine dehydrogenase	Oxidative stress	Oxidative metabolism of purines	Linked to nitroso-redox balance, also possible plasma marker	[104]
69	NOS1	Nitric oxide synthase 1	Oxidative stress	Synthesizing nitric oxide from L-arginine	Inhibition possibly linked to protection of diastolic dysfunction	[105, 106]
14,19	GSTZ1, GSTT1	Glutathione-S-transferases	Oxidative stress	Catalyzing the conjugation of the reduced form of glutathione to xenobiotic substrates for the purpose of detoxification	Related to oxidative stress in cardiac tissue	[107, 108]

### Corroboration of HFpEF gene candidates in the transcriptome of a murine HFpEF model

After translating the comorbidity profile of HFpEF patients to an associated genetic profile, the functional relevance of this predicted profile remained unclear. We hypothesized that the relevance of the gene predictions could be suggested by transcriptional dysregulation in myocardial tissue of HFpEF. However, human molecular data of HFpEF is sparse and mechanistic insights are typically derived from mouse models [3]. Hence, we collected myocardial bulk transcriptomics from a murine HFpEF model, induced via high-fat diet and nitric oxide synthase inhibitor N[w]-nitro-l-arginine methyl ester (L-NAME) treatment [36] (Fig. 5C, Additional file 1: Supplementary Methods).

First, we confirmed important HFpEF phenotype characteristics including preserved ejection fraction, increased body weight, blood glucose levels, and blood pressure as well as diastolic dysfunction (increased E/e') (*n*_CT_ = 4, *n*_HFpEF_ = 4, Student’s *t*-test, *p* < 0.05, Additional file 1: Fig. S12*)*.

Second, we assessed transcriptomic changes in HFpEF via differential gene expression analysis (Additional file 1: Fig. S13A-D). Upregulated genes confirmed processes involving fibrosis and metabolic stress (Additional file 1: Fig. S13C, D).

After confirming the phenotypic and molecular resemblance of the HFpEF model, we investigated gene expression dysregulation of the comorbidity-based gene profiles by performing enrichment analysis of the HFpEF and HFrEF gene predictions (Fig. 5D, Additional file 1: Fig. S13E). We found that the top 50 to 100 predicted HFpEF genes displayed significant enrichment in overexpressed genes in the murine HFpEF model, while the HFrEF predicted genes were not enriched (*p*-value < 0.05, Fig. 5E, Additional file 1: Fig. S13E). Fibrosis-related genes like LOX, SMAD9, and PTHL and hypertrophy-related genes like GATA5, GATA3, and MYH7 could be recovered together with XDH, among others. This suggested that the genetic profile derived from human HFpEF comorbidities associated with relevant gene expression dysregulation during myocardial remodeling in murine HFpEF.

## Discussion

In this study, we provided a retrospective systems level analysis of comorbidities in a large cohort of HF patients. We derived clinically relevant insights by comparing comorbidity profiles between HFpEF and HFrEF patients and biological insights by defining genes associated with HFpEF and HFrEF comorbidity profiles.

Patient clustering has been previously shown to yield novel subgroups of HFpEF defined by multivariate similarity [109–111]. In contrast, the clustering of features (i.e., comorbidities) can inform about patterns of co-occurring disease groups. Our study demonstrated that this approach can be useful to interpret comorbidity profiles: the aggregation of co-occurrence patterns of diseases can help to organize illness into different levels of clinical concepts like organs (DC7—gastrointestinal tract), illness severity (DC5—intensive care), or disease categories (DC8—cancer). This aggregation via network clustering may also reduce multiple testing burdens and provide insights into the relevance of low prevalence diseases where comparisons for a single disease may be problematic.

In the patient classifier, HFpEF was characterized by a larger number of comorbidities with lesser emphasis on cardiac disorders. This supports the hypothesis of HFpEF as a comorbidity-driven systemic syndrome [112, 113]. We found that hypertensive heart disease was the most discriminant feature for HFpEF, which has been viewed as a major etiology for diastolic HF involving cardiac hypertrophy and myocardial stiffness [114, 115]. In contrast, ischemic etiologies including myocardial infarction characterized HFrEF consistent with other studies [116].

We identified more novel disease associations with HFpEF such as neoplastic diseases including breast cancer. HF related hospitalizations in breast cancer survivors have been recently associated more with HFpEF than with HFrEF [117], though the reasons for this remain incompletely elucidated [118]. The association to other cancerous diseases remains largely unexplored and should be addressed in future studies. Another interesting aspect of the HFpEF comorbidity profile was the high similarity to DC6, which contained rheumatic, osteologic, and mental diseases. Systemic inflammatory diseases could be a driving factor for HFpEF and rheumatic disease could constitute a pathophysiologic linkage [112, 119–121]. Bone mineralization also has been reported to be lowered in HFpEF patients [122] and is a symptomatic link to postmenopausal endocrinology [123]. While mental health has been studied in the context of HF extensively, differences between HFpEF and HFrEF are largely unexplored. The joint clustering of these disease complexes and their similarity to female patients provides a potential link between female sex and HFpEF. Future work should further explore these relationships.

HFpEF and HFrEF clearly displayed distinguishable comorbidity profiles. By contrast, HFmrEF, introduced as a unique form of HF in 2016 [124], appeared to be a combination of attributes from HFrEF and HFpEF. Thus, from the comorbidity perspective, it may be a transitional state instead of a unique syndrome as suggested before [125].

We predicted an associated genetic profile from data-driven HFpEF comorbidity profiles. This genetic profile indicates that HFpEF comorbidities are associated with recurrent patterns of genes involved in fibrosis, inflammation, cell differentiation, metabolism, and oxidative stress. As an example, the glutathione-S-transferases, NOS1 and Xanthine dehydrogenase (XDH), were identified by our network. XDH catalyzes the rate limiting step in purine metabolism producing uric acid [126] and previous literature supports both the role of serum uric acids in HF [104] and plasma XDH activity as relevant for adverse clinical outcomes in HFpEF [127]. Nitric oxide synthase (NOS) has been proposed to contribute to endothelial dysfunction in HFpEF [105, 106], and NOS1 inhibition was recently associated with recovery of diastolic dysfunction in a murine model resembling HFpEF [128]. Glutathione-S-transferases (GSTM1, GSTT1, GSTZ1) are antioxidant enzymes and polymorphisms of these genes have been reported as potentially relevant to HF and diastolic dysfunction [107, 108]. This group of genes could constitute crucial gene candidates involved in comorbidity-based HFpEF pathophysiology.

In general, HFpEF is likely to be a disease in which multiple genes and pathways contribute to the spectrum of phenotypes. Therefore, instead of using the disease–gene prediction to identify and validate individual genes, we have corroborated the overall effect of a spectrum of identified genes in murine gene expression data. While this provided additional evidence for the relevance of comorbidity-based gene prediction, further experimental validation is necessary to explore the functional role and reproducible validity of candidate genes. In real-world populations, it is likely that the genetic heterogeneity of the HFpEF syndrome will be influenced by the specific comorbidities that are well represented in each population. In previous disease–gene prediction studies, gene prediction was performed either by selecting multiple seed genes or single seed diseases [129, 130]. We propose that our approach for gene inference based on data-driven comorbidity profiles might be suitable for systemic syndromes where multimorbidity plays an important role like HF and especially HFpEF.

In addition, several data resources were generated in this study: (i) the HFpEF gene predictions, (ii) HFhetnet, and (iii) murine HFpEF transcriptome to help facilitate future efforts to understand HFpEF-related pathophysiology and benefit the research community.

This study had important strengths and was subject to several limitations. An important strength of this study is that we analyzed clinical care data, which is a real-world representation of patients and therefore allowed us to perform a data-driven analysis of comorbidities in this patient population. However, as a result, this analysis is limited to the information captured (i) in our hospital system and (ii) at the hospital visits of a patient. Therefore, obtained results could be subject to some common biases found in medical record-derived data, such as non-random interaction with the health care system resulting in some patient populations having more data than others [131], incomplete documentation [132], selection bias [133], missing data, and lack of documentation of potential confounders [134]. In addition, possible non-observed confounders like socioeconomic status or health-related behavior could not be taken into account due to lack of documentation in the medical record. We determined subtypes using LvEF, which can be error prone [135] and might not fully provide a sufficient criterion for the HFpEF diagnosis [136]. Patients with more serious conditions will tend to visit a tertiary health care provider more often and thus could be overrepresented. In our study, at a tertiary care center with a focus on cardiomyopathy, this seemed to affect the contrast between HFrEF and HFpEF, as HFrEF patients had higher intubation prevalence and DC 5 similarity. This may also have contributed to differences between this study population and other reports of HFpEF population characteristics. However, given the known heterogeneity of HFpEF and HFrEF [109–111], we believe these differences are plausible and a more granular approach to study HFpEF subtypes could be necessary to address inconsistent patient characteristics [137]. Another limitation of our study is the use of ICD-10 codes to capture comorbidities. Different ICD-10 codes are known to have different predictive value for disease, and therefore, it is likely that some diseases are over- or underrepresented in our data [138]. Moreover, we performed a cross-sectional analysis and therefore did not consider the timing and sequence of comorbidities when generating comorbidity profiles.

Given these limitations, future studies are necessary to address the generalizability of our findings to other HF populations and to delineate different disease trajectories by considering the time of events. Nevertheless, our study recapitulated known HF comorbidity patterns, as discussed above, that could substantiate more novel comorbidity patterns identified in this work.

Many open questions remain regarding HFpEF pathophysiology and genetics [16]. Interdisciplinary and translational approaches are needed to account for the cross-organ disease involvement that is suggested to be critical in HFpEF. The increasing abundance of routine clinical care data and novel approaches like network medicine can provide novel insights and guidance for future experimental approaches.

## Conclusions

In our study, we found evidence for greater diversity of comorbidity profiles in patients with HFpEF compared to HFrEF. We further identified nine co-occurring disease groups which capture differences of disease prevalence regarding age, sex, and HF subtype. Here, we find that multimorbidity in HFpEF extends to disease clusters beyond typical HF comorbidities and includes rheumatoid, neoplastic, and gastrointestinal diseases. We further provided a biological interpretation of the HFpEF comorbidity profile, capturing overexpressed gene programs observed in murine HFpEF models. Oxidative stress, hypertrophy, cell differentiation, and fibrosis-related genes are recurrent patterns in genes associated to comorbidities of HFpEF and could constitute a link for the comorbid relationships of HFpEF resulting in a multiorgan disease state. Thus, our work highlights that comorbidity profiles are an important characteristic of HFpEF patients and should be incorporated into both clinical and genomic approaches to the study of HFpEF.

## Supplementary Information

**Additional file 1: Supplementary Methods and Supplementary Figures S1-13. Methods: **Processing of Comorbidities  and Other Clinical Predictors Patient classifier Comorbidity profile assignment compared for effects of age, sex, time to HF diagnosis and time of recording Comorbidity network clustering Disease network comparison Disease prediction metrics Heart failure gene sets. **Fig. S1.** ICD10 code mapping. **Fig. S2.** Patient classifier training.**Fig. S3.** Parameter estimates from the patient classifier.**Fig. S4.** Time to HF and time of comorbidity profile assignment.**Fig. S5.** Comparison of comorbidities between HFpEF and HFrEF cohort.**Fig. S6.** HFnet overview.**Fig. S7.** Comparison of disease networks.**Fig. S8.** Comparison of centralities.**Fig. S9.** Comparison of clustering algorithms in the HFnet.**Fig. S10.** Leave-one-out cross validation of disease gene prediction.**Fig. S11.** HF subtype gene prediction.**Fig. S12.** L-NAME/HFD Phenotype data.**Fig. S13.** Myocardial gene expression in L-NAME/HFD. L-NAME/ HFD mouse model and RNA sequencing.

**Additional file 2: Supplementary Table 1. **Manual disease code mappings

**Additional file 3: Supplementary Table 2. **Centrality of nodes in HFnet

**Additional file 4: Supplementary Table 3. **Disease Cluster Composition of the HFnet

**Additional file 5: Supplementary Table 4. **Edge list of HFhetnet

**Additional file 6: Supplementary Table 5. **Predicted HFpEF and HFrEF genes

## Data Availability

All code from this analysis is available at github.com/janlanzer/hf_comorbidity. Clinical data could not be deposited for patient privacy reasons. Model outputs such edge tables of HFnet and HFhetnet are available in table format in Supplementary Tables. Murine RNA-sequencing data has been deposited in NCBI’s Gene Expression Omnibus (GEO) and is accessible through GEO Series accession number GSE225557.

## Abbreviations

AUROC: Area under the receiver operating characteristic
BH: Benjamini–Hochberg
DC: Disease clusters
GO: Gene Ontology
HF: Heart failure
HFnet: Heart failure comorbidity network
HFhetnet: Heart failure multilayer heterogeneous network
HFmrEF: Heart failure with mid-range ejection fraction
HFpEF: Heart failure with preserved ejection fraction
HFrEF: Heart failure with reduced ejection fraction
ICD: Implantable cardioverter defibrillator
ICD-10: International classification of disease, version 10
LvEF: Left ventricular ejection fraction
MCA: Multiple correspondence analysis
NTproBNP: N-terminal pro b-type natriuretic peptide
PheCodes: Phenome-wide association scan codes
PPI: Protein–protein interaction
UMLS: Unified Medical Language System
