# Supplementary material for: A network medicine approach to study comorbidities in heart failure with preserved ejection fraction
Source: BMC Med. 2023 Jul 24;21:267. doi: 10.1186/s12916-023-02922-7 (PMC10367269; doi:10.1186/s12916-023-02922-7)
Supplement: Supplementary file 1 — Additional file 1: Supplementary Methods and Supplementary Figures S1-13. Methods: Processing of Comorbidities and Other Clinical Predictors Patient classifier Comorbidity profile assignment compared for effects of age, sex, time to HF diagnosis and time of recording Comorbidity network clustering Disease network comparison Disease prediction metrics Heart failure gene sets. Fig. S1. ICD10 code mapping. Fig. S2. Patient classifier training.Fig. S3. Parameter estimates from the patient classifier.Fig. S4. Time to HF and time of comorbidity profile assignment.Fig. S5. Comparison of comorbidities between HFpEF and HFrEF cohort.Fig. S6. HFnet overview.Fig. S7. Comparison of disease networks.Fig. S8. Comparison of centralities.Fig. S9. Comparison of clustering algorithms in the HFnet.Fig. S10. Leave-one-out cross validation of disease gene prediction.Fig. S11. HF subtype gene prediction.Fig. S12. L-NAME/HFD Phenotype data.Fig. S13. Myocardial gene expression in L-NAME/HFD. L-NAME/ HFD mouse model and RNA sequencing. [file 12916_2023_2922_MOESM1_ESM.pdf]

Supplementary Data

A network medicine approach to study comorbidities in heart failure with preserved ejection fraction

Jan D. Lanzer, Alberto Valdeolivas, Mark Pepin, Hauke Hund, Johannes Backs, Hans-Christoph Friederich, Norbert Frey, Jobst H. Schultz, Julio Saez-Rodriguez, Rebecca T. Levinson

1. Supplementary Methods **2**
Processing of Comorbidities and Other Clinical Predictors 2
Patient classifier 3
Comorbidity profile assignment compared for effects of age, sex, time to HF diagnosis and time of recording 4
Comorbidity network clustering 5
Disease network comparison 6
Disease prediction metrics 7
Heart failure gene sets 8
L-NAME/ HFD mouse model and RNA sequencing 9
2. Supplementary Figures **12**
Fig. S1. ICD10 code mapping. 12
Fig. S2. Patient classifier training. 13
Fig. S3. Parameter estimates from the patient classifier. 15
Fig. S4. Time to HF and time of comorbidity profile assignment. 16
Fig. S5. Comparison of comorbidities between HFpEF and HFrEF cohort. 17
Fig. S6. HFnet overview. 18
Fig. S7. Comparison of disease networks. 19
Fig. S8. Comparison of centralities. 20
Fig. S9. Comparison of clustering algorithms in the HFnet. 22
Fig. S10. Leave-one-out cross validation of disease gene prediction. 23
Fig. S11. HF subtype gene prediction. 24
Fig. S12. L-NAME/HFD Phenotype data. 25
Fig. S13. Myocardial gene expression in L-NAME/HFD. 26

1. Supplementary Methods

Processing of Comorbidities and Other Clinical Predictors

Comorbidities were recorded in ICD-10 format. To reduce features and summarize similar diseases we mapped ICD-10 codes to PheWas Codes. We accessed "https://phewascatalog.org/" in October 2021 and downloaded "PheCodeMap 1.2". From a total of 7817 unique ICD-10 codes in our dataset, 3030 could be mapped directly to PheCodes. To further improve this coverage we performed an additional stepwise mapping: the 4787 ICD-10 codes that could not be mapped were shortened to 4 characters (e.g. N18.89 was shortened to N18.8) and mapped again to PheCodes. Unmapped ICD-10 codes after this step were shortened to 3 characters (e.g. N18.8 shortened to N18) and mapped again to PheCodes, resulting in a total of 6676 mapped ICD-10 codes. Most frequent ICD-10 codes that were not mapped to PheCodes included codes from the Z-chapter that were considered outside the area of interest of this study and were discarded after this step. Total coverage of the mapping is 85% (mapped ICD-10 codes/unmapped ICD-10 codes) resulting in 1481 unique PheCodes (Supp Fig. 1A). We calculated patient frequencies in the HF cohort for these PheCodes and only analyzed PheCodes with at least 50 patients, reducing the features to 569 PheCodes (Supp Fig. 1B).

OPS codes were used to determine heart transplant patients (5-375, together with ICD-10 code Z94.1), patients that were intubated (8-701, 8-704, 8-706, 8-852), patients that underwent percutaneous coronary intervention (8-83) and patients that underwent anit-arrhythmic device implantation (5-378, 5-377).

The Elixhauser index was calculated per patient using the comorbidity R-package [19]. Laboratory values for low-density lipoprotein (LDL), high-density lipoprotein (HDL), triglycerides, and total cholesterol were obtained. For each measure minimal quality control was performed to remove values that were negative or extreme (values greater than 400 for LDL, 150 for HDL, 600 for triglycerides were removed). Heights and weights were also obtained for individuals in the HF population. After cleaning to remove implausible values, heights and weights on the same day were used to calculate body mass index (BMI). BMIs greater than 70 were excluded, and a median BMI was calculated for each patient.

Patient classifier

Random forest and regularized logistic regression were fit to predict HFpEF and HFrEF cohort labels on 569 PheCodes. For hyperparameter tuning, we performed 10 fold cross validation of 90-10% training -test splits and selected hyperparameter values yielding highest mean AUROC. Hyperparameters include the ratio of L1/L2 regularization and penalty for elastic net and number of variables (mtry) and number of trees in random forest. The highest achieved mean AUROC is reported as an estimate for the model test error (Supp Fig. 2A, B). For model interpretation, we then performed a model fit on the full data using the best estimated hyperparameters. From these models we analyzed feature importances (random forest) and parameter estimates (elastic net) (Fig. S2C).

Comorbidity profile assignment compared for effects of age, sex, time to HF diagnosis and time of recording

Age and Sex

We selected the comorbidity profiles of HFpEF and HFrEF (i.e. top 100 comorbidities from the patient classifier) and tested each in an independent logistic regression model while including age and sex as covariates for association with HF subtype label (HFpEF/HFrEF ~ comorbidity + sex + age). We fit these models on the full cohort data and found that the coefficients assigned to the comorbidities to be consistent with the patient classifier assignments (Supp Fig. 4A, column "full data").

Time to HF diagnosis

We investigated whether the comorbidity profiles of HFpEF or HFrEF were different in regard to the time point of the patient's first HF diagnosis. We calculated the time to HF diagnosis in months and found that most comorbidities were recorded within a year of the first HF diagnosis, which is most likely related to the nature of routine clinical care data from a tertiary care provider than with the true time point of comorbidity occurence. Nevertheless, HFpEF patients received their comorbidities later than HFrEF patients (Wilcoxon's test $p<0.05$) (Fig. S4B).

We next subset the data for each patient to his comorbidities recorded at least 6 months before (pre HF) or after (post HF). On these subsets we again fitted the logistic regression model (HFpEF/HFrEF ~ comorbidity + sex + age) and found a conserved assignment of comorbidities. This indicated that comorbidity

assignment to HFpEF or HFrEF was rather independent of the time point of HF diagnosis (Fig. S4A).

Date of comorbidity assignment

Recording of comorbidities is subject to clinical practice that may change over time. We thus compared the dates of comorbidity assignments and found that HFpEF patients recorded more recent diagnosis compared to HFrEF patients (Supp Fig. 4C). To investigate if this difference in time of recording impacted our comorbidity profile assignment we stratified our observation window into three time blocks. We again fit the logistic regression models (HFpEF/HFrEF ~ comorbidity + sex + age) for each time block separately and observed that the assignment of most comorbidities was not dependent on the observation window (Supp Fig. 4A).

Comorbidity network clustering

To identify disease communities (DCs) within the network we applied different clustering algorithms. We assumed that if different clustering algorithms detect similar structures, these structures could be more reliable. We compared different cluster algorithms based on the shared information between the assigned cluster labels (normalized mutual information and adjusted Rand Index) as well as modularity scores and module size and number. The Leiden algorithm[20] achieved the highest adjusted Rank index and normalized mutual information scores when compared to other tested algorithms (Fig. S7A-E). We then tested different resolution parameters of the Leiden algorithm and selected

a parameter of 1.1 that yielded multiple clusters and maintained a high network modularity score and high normalized mutual information values (Fig. S7F).

To represent patients based on their disease profile similarity with the network's DCs, we calculated Jaccard indices for each patient and DC and performed unpaired, two-sided Wilcoxon's rank sum test to test for differences between patient cohorts.

Disease network comparison

We compared the Heart Failure Comorbidity Network (HFnet) with two other disease networks. We downloaded network data from Morbinet (https://shiny.odap-ico.org/morbinet, accessed October 2021) [11], using a threshold of OR>1 and fisher p value of p<0.01 and mapped the ICD-10 disease ontology to PheCodes. As a second network, we built a phenotypic disease network, where two diseases are connected, if they share a similar phenotype based on the human phenotype ontology (HPO) [29]. To construct this network, we downloaded the HPO from https://hpo.jax.org/app/ (accessed October 2021) and mapped disease ontologies to Phecodes. Disease similarity was calculated with Lin's methods implemented in the *OntologySimilartiy* R-package [21]. The full distance matrix resulting from the ontology similarity was used to create a fully connected network with edge weight representing ontology similarity. From this network we extracted the backbone [22] with the implementation in the *corpustools* R-package. This backbone extraction is based on an assumed null distribution of local edge weights where based on an alpha level (here 0.05) edges can be extracted that are unlikely to fall into that distribution.

For network comparison, Jaccard indices of Nodes and Edges were calculated (Fig. S8B,C) on different subgraphs of HFnet, Morbinet and HPOnet. DeltaCon measures graph distance of two graphs with the same set of nodes. It first calculates pairwise node affinities via Fast Belief Propagation in each network and in the second step measures distance between both affinity matrices via root euclidean distance [23]. We subsetted HFnet nodes to match Morbinet and HPOnet and calculated DeltaCon distances. Furthermore, we rewired each HFnet subset five times with an increasing probability and computed DeltaCon similarity with the original HFnet (Supp. Fig 8D).

Disease prediction metrics

The mapping of UMLS codes in DisGeNET to ICD-10ICD10 and PheCodes led to few diseases having assigned the same or highly similar gene sets. To avoid overfitting by disease neighbors with highly similar gene-disease pairs, we calculated a binary distance (Jaccard index) between all DisGeNET gene sets. For the internal validation of a disease-gene prediction, we then removed all other gene associations of diseases that were highly similar to the target gene set (jaccard distance cutoff 0.5) (Fig. S9B).

To evaluate gene prediction success, we used three different metrics: median rank ratio, AUROC and AUCPR.

For the median rank ratio, we calculated the median rank of the target gene set in the RW ranking and divided this rank by the total length of the ranking. This metric is close to 0 if the genes are located towards the top, and close to 1 if they are located close to the bottom of the ranking.

AUROCs and AUCPRs were calculated with the R-package *pROCroc*. Each target gene was considered as a true positive, others were true negatives and the assigned RW probabilities were used to calculate area under the ROC and PR curves. AUC-PRs tend to be very low, due to the high number of non-target genes in the top of the ranking that leads to a drop in precision. This in part is wanted for disease gene prediction, as these true negative genes could rather be unknown potentially relevant candidates. AUROCs can be inflated when small gene sets are recovered in the top of the ranking. We assessed this bias by correlating gene set size to performance metric (Fig. S9B).

To assess whether link prediction performance is dependent on HFnet topology, we repeated the internal validation process, described above with rewired edges in the HFnet. Network rewiring was performed with the *igraph* R-package, using *rewire()* and *each_edge()* functions.

Heart failure gene sets

General HF associated genes were curated by collecting genes from various resources: We selected prior knowledge sources including 1) DisGeNet genes associated to Heart Failure with confidence score >0.29; 2) Literature curated [24], 3) Kegg disease database, dilated cardiomyopathy related pathways [34] and data driven resources including 4) Cardiovascular Disease Knowledge portal, top common variants for Heart Failure [30]; 5) Cardiovascular Disease Knowledge portal, top single variants for Heart Failure [30] (https://cvd.hugeamp.org/, accessed August 2022); 6) ReHeaT top 500 conserved genes from end stage heart failure meta-analysis [25, 33]. 7) PheWAS gene sets associated with Heart

Failure ($p<0.05$, Odds ratio>1) [31, 32] (https://phewascatalog.org/ , accessed August 2022).

L-NAME/ HFD mouse model and RNA sequencing

Animal Use Approval and Ethics. Animal model experiments were performed in accordance with the European Community guiding principles in the care and use of animals (2010/63/UE, 22 september 2010) as approved by by the Niedersächsisches Landesamt für Verbraucherschutz und Lebensmittelsicherheit (G42-21, Baden-Württemburg, Germany).

Animal Model. 12-week-old C57BL/6N male mice were bred at the Universitätsklinikum Heidelberg Klinisch Experimenteller Bereich mouse facility. Mice were given *ad libitum* access to the combination of high-fat chow and N(ω)-nitro-L-arginine methyl ester (L-NAME, 0.5 g/L, pH = 7.4, Sigma-Aldrich) (HFD-LNAME) or control chow diet for a duration of 9 weeks (n = 4), as previously described (**Fig. S11**) [36]. Female mice were not used owing to their protection against diastolic dysfunction in response to HFD-LNAME feeding [37]. All cohorts of mice were group-housed (≤3 mice/cage) on a 12:12-h light-dark cycle from 06:00 to 18:00 at 25 ± 1°C and constant humidity with *ad libitum* access to either standard chow (2916, Teklad) or HFD (58.0 kcal% fat, D12492) and water.

RNA-sequencing. Following 9-week treatment with HFD-LNAME, total RNA was isolated from left ventricular mouse heart tissue using TRIzol™ Reagent (ThermoFisher, USA), subsequently tested for quality using a Bioanalyzer 2100 (Agilent) to ensure RIN > 9. Total RNA was depleted from ribosomal RNA, polyA-enriched, fragmented, and paired-end sequenced at the European

Molecular Biology Laboratory (EMBL). Alignment of reads were mapped to the latest *Ensembl* C57BL/6NJ annotation (c57bl_6NJ_107.gtf) to quantify transcripts. Alignment was performed using *STAR* (2.7.10a) [38].

2. Supplementary Figures

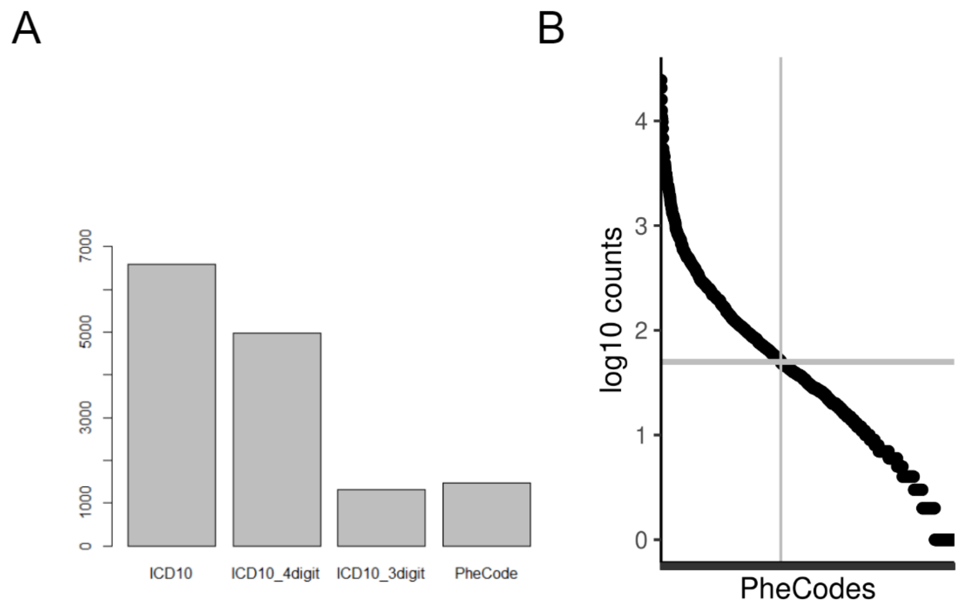

Fig. S1. ICD10 code mapping.
A) Number of features recorded in the general HF cohort. PheCodes and 3-letter ICD10-codes reduce the feature space to a comparable feature size. C) Overview of all recorded PheCodes and their frequency (log10 transformed). PheCodes with a prevalence of at least 50 patients (horizontal grey line) resulted in 569 Phecodes (vertical grey line). These PheCodes were used in all downstream analysis.

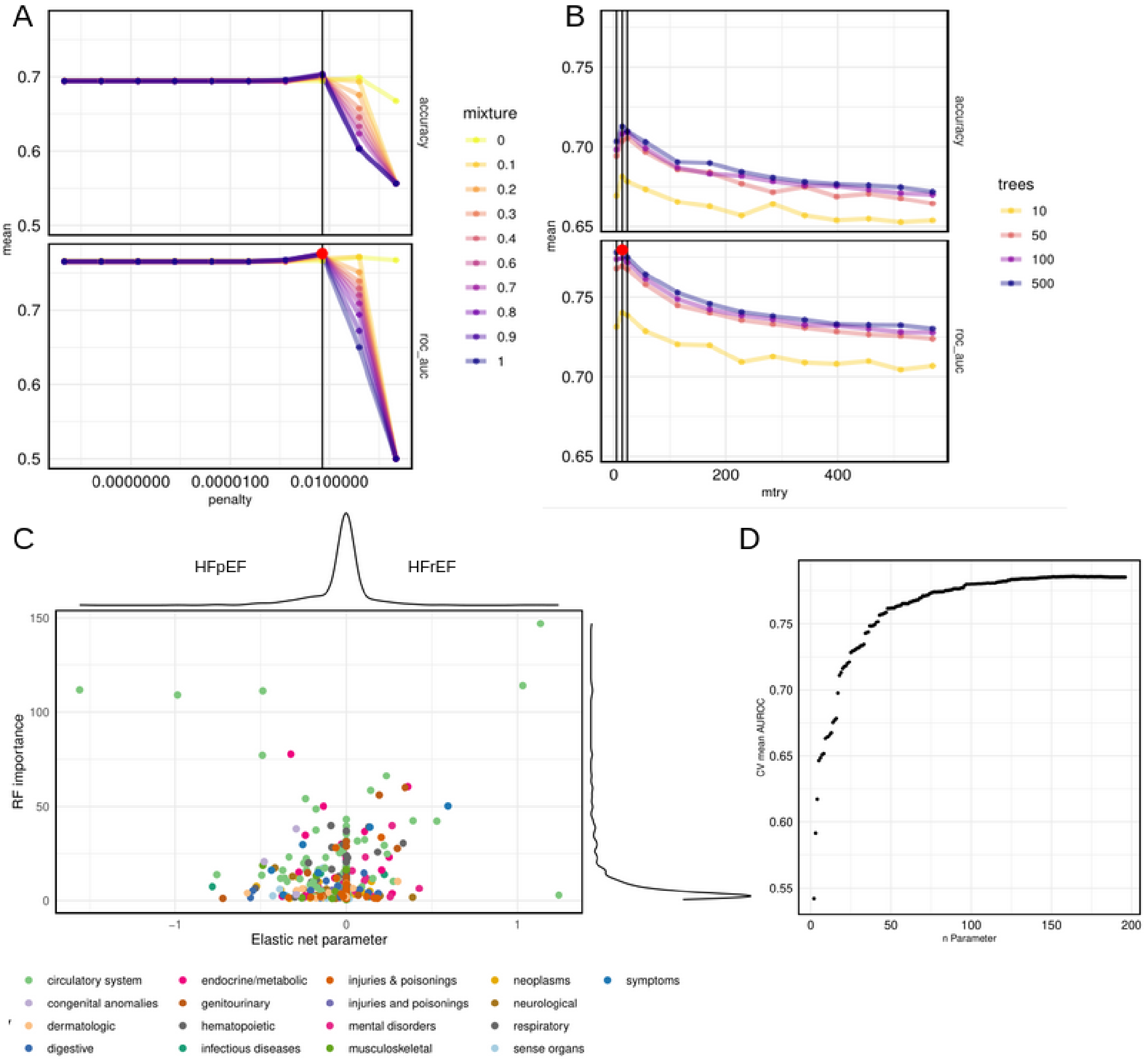

Fig. S2. Patient classifier training.
A) Hyperparameter tuning for the elastic net model. Mixture of L1 and L2 penalty (color) and penalty value (x-axis) are compared by accuracy and AUROC. Each hyperparameter combination was assessed via 10-fold CV-splits. B) Hyperparameter tuning for random forest model. Number of trees (color) and mtry (x-axis) are compared by accuracy and AUROC. Each hyperparameter combination was assessed via 10-fold CV-splits. C) Comparison of random forest feature importance (y-axis) with Elastic net coefficient estimates (x-axis). D) Forward selection training of L1- regularized logistic regression by stepwise including parameters to the model (x-axis) and estimating a 10-fold CV AUROC (y-axis).

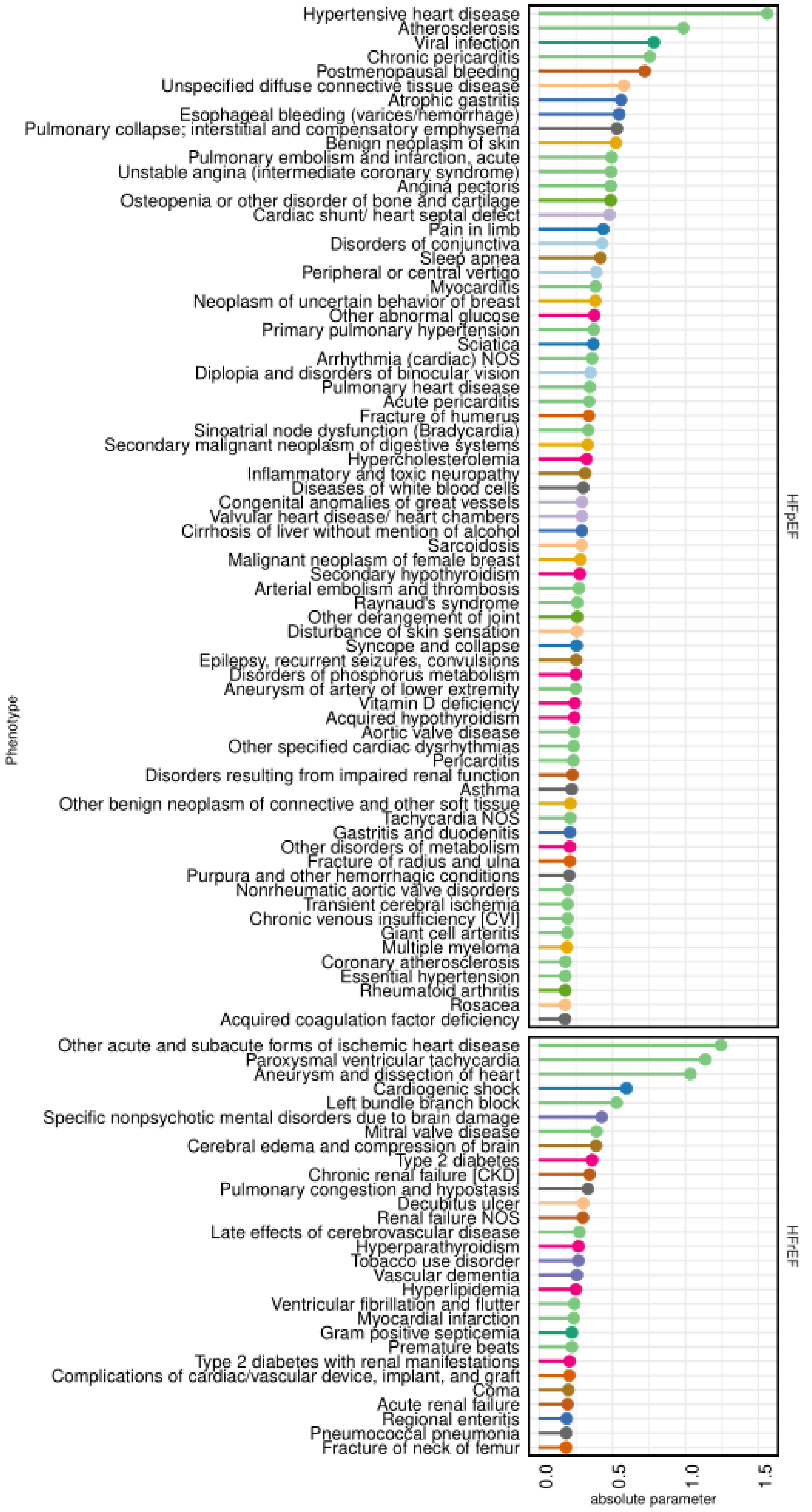
Hypertensive heart disease
Atherosclerosis
Viral infection
Chronic pericarditis
Postmenopausal bleeding
Unspecified diffuse connective tissue disease
Atrophic gastritis
Esophageal bleeding (varices/hemorrhage)
Pulmonary collapse; interstitial and compensatory emphysema
Benign neoplasm of skin
Pulmonary embolism and infarction, acute
Unstable angina (intermediate coronary syndrome)
Angina pectoris
Osteopenia or other disorder of bone and cartilage
Cardiac shunt/ heart septal defect
Pain in limb
Disorders of conjunctiva
Sleep apnea
Peripheral or central vertigo
Myocarditis
Neoplasm of uncertain behavior of breast
Other abnormal glucose
Primary pulmonary hypertension
Sciatica
Arrhythmia (cardiac) NOS
Diplopia and disorders of binocular vision
Pulmonary heart disease
Acute pericarditis
Fracture of humerus
Sinoatrial node dysfunction (Bradycardia)
Secondary malignant neoplasm of digestive systems
Hypercholesterolemia
Inflammatory and toxic neuropathy
Diseases of white blood cells
Congenital anomalies of great vessels
Valvular heart disease/ heart chambers
Cirrhosis of liver without mention of alcohol
Sarcoidosis
Malignant neoplasm of female breast
Secondary hypothyroidism
Arterial embolism and thrombosis
Raynaud's syndrome
Other derangement of joint
Disturbance of skin sensation
Syncope and collapse
Epilepsy, recurrent seizures, convulsions
Disorders of phosphorus metabolism
Aneurysm of artery of lower extremity
Vitamin D deficiency
Acquired hypothyroidism
Aortic valve disease
Other specified cardiac dysrhythmias
Pericarditis
Disorders resulting from impaired renal function
Asthma
Other benign neoplasm of connective and other soft tissue
Tachycardia NOS
Gastritis and duodenitis
Other disorders of metabolism
Fracture of radius and ulna
Purpura and other hemorrhagic conditions
Nonrheumatic aortic valve disorders
Transient cerebral ischemia
Chronic venous insufficiency [CVI]
Giant cell arteritis
Multiple myeloma
Coronary atherosclerosis
Essential hypertension
Rheumatoid arthritis
Rosacea
Acquired coagulation factor deficiency
HFpEF
Other acute and subacute forms of ischemic heart disease
Paroxysmal ventricular tachycardia
Aneurysm and dissection of heart
Cardiogenic shock
Left bundle branch block
Specific nonpsychotic mental disorders due to brain damage
Mitral valve disease
Cerebral edema and compression of brain
Type 2 diabetes
Chronic renal failure [CKD]
Pulmonary congestion and hypostasis
Decubitus ulcer
Renal failure NOS
Late effects of cerebrovascular disease
Hyperparathyroidism
Tobacco use disorder
Vascular dementia
Hyperlipidemia
Ventricular fibrillation and flutter
Myocardial infarction
Gram positive septicemia
Premature beats
Type 2 diabetes with renal manifestations
Complications of cardiac/vascular device, implant, and graft
Coma
Acute renal failure
Regional enteritis
Pneumococcal pneumonia
Fracture of neck of femur
HFrEF
Phenotype
0.0
0.5
1.0
1.5
absolute parameter

Fig. S3. Parameter estimates from the patient classifier.
The parameters are the absolute fitted values of the coefficients in the elastic net model for each comorbidity of the patient classifier separated by association to HFpEF (top) or HFrEF features (bottom). Colors indicate disease category using the same color legend as in panel B.

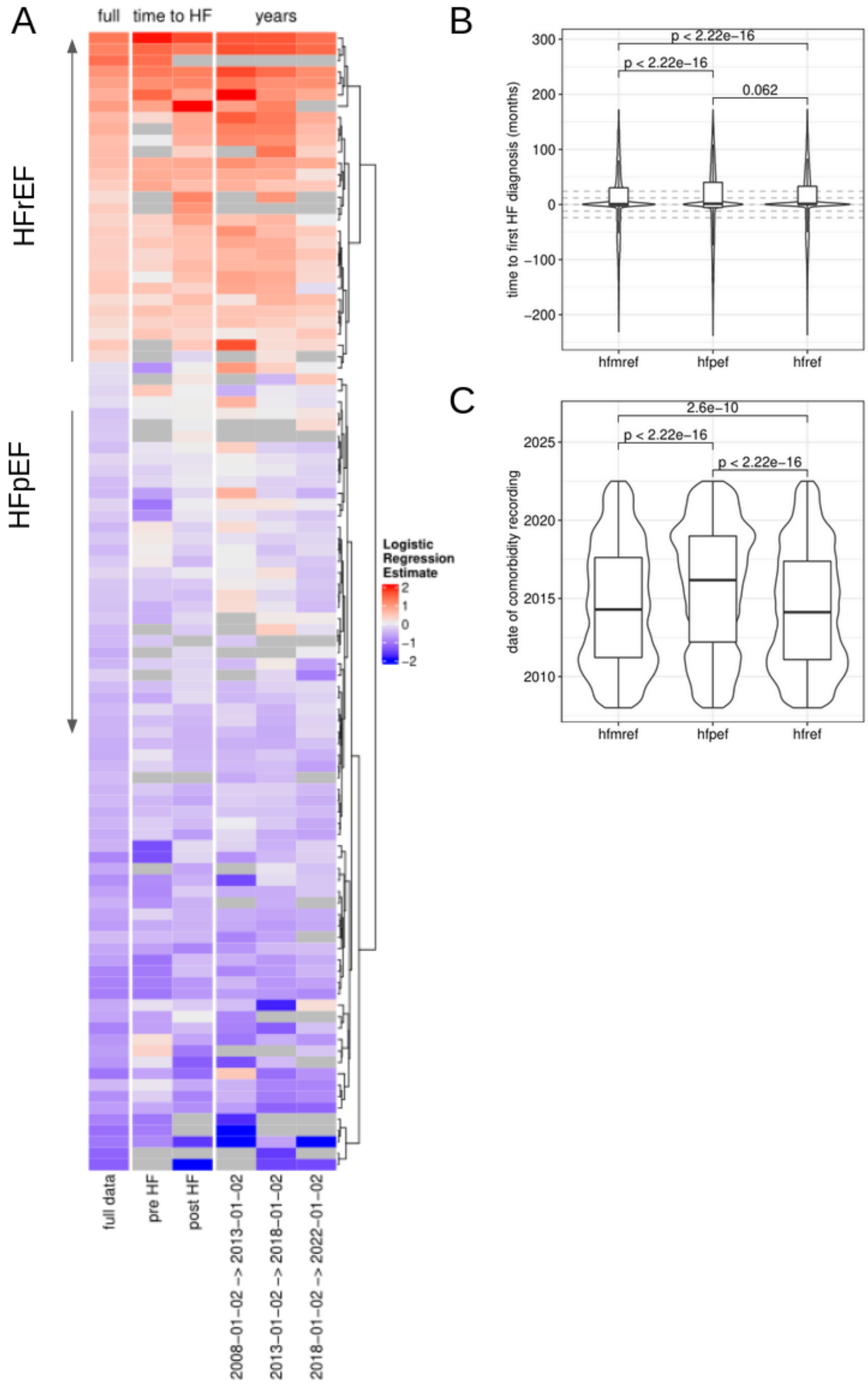

Fig. S4. Time to HF and time of comorbidity profile assignment.
A) Comparing the comorbidity assignment to HF subtypes (y-axis) in different data subsets (x-axis). Logistic Regression models were fit without regularization for each comorbidity separately to predict HF subtype labels (HFpEF/HFrEF ~ comorbidity + age + sex). First column contains the comorbidity estimates from the full data set. Second block displays comorbidity estimates form the data subset to comorbidities with earliest diagnosis at least six months before (pre HF) or six months after the first HF diagnosis (post HF). The third block displays comorbidity estimates from data subset to three different observation windows.
B) Distribution of the time in months between earliest comorbidity diagnosis

and earliest HF diagnosis compared between HF cohorts (p-value, unpaired two-sided Wilcoxon test). Dotted gray lines are each 12 months apart. C) Distribution of recording dates of comorbidities in HF subtype cohorts over the observation window (2008-2021) (p-value, unpaired two-sided Wilcoxon test).

A

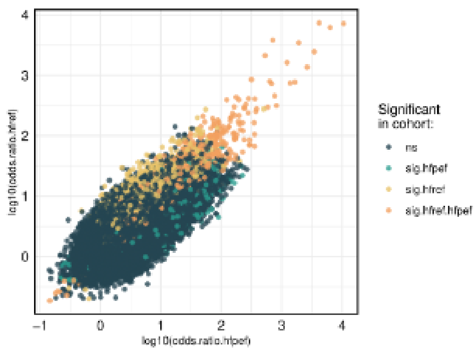

C

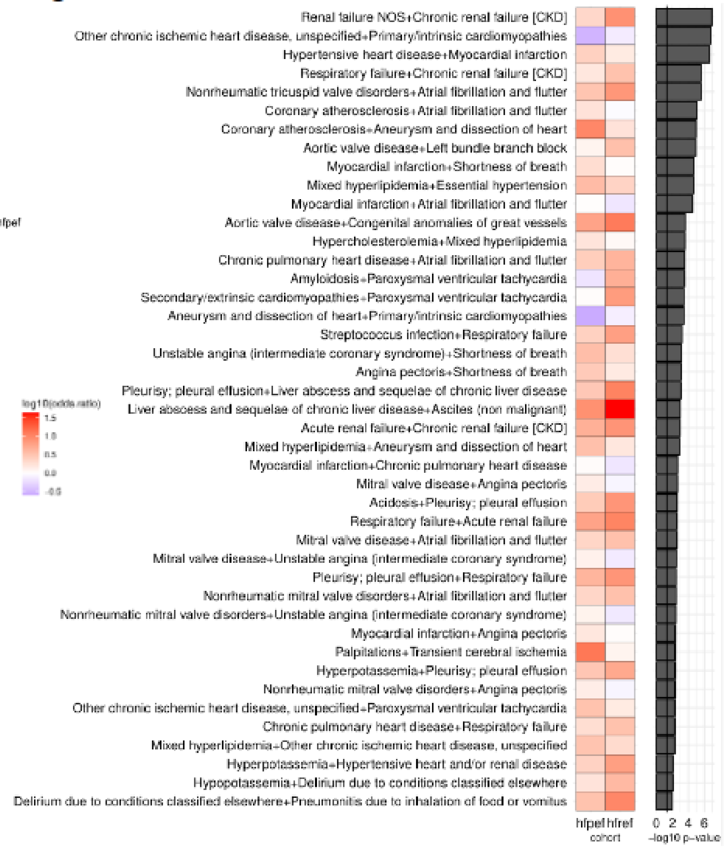

B

	HFpEF Cohort	
HFrEF Cohort	sig. disease pair	ns. disease pair
sig. disease pair	4.2%	4.9%
ns. disease pair	2%	88.9%

Fig. S5. Comparison of comorbidities between HFpEF and HFrEF cohort. A) Pairwise disease odds ratios and Fisher's exact test p-values (with BH correction) were calculated for HFpEF and HFrEF patient cohorts separately. Pearson correlation of odds ratios between both cohorts was ~1 with p<0.01. B) Comparison of all tested disease pairs at fisher test p<0.0001. C) Breslow dayes test for homogeneity of odds ratios was applied and significant disease pairs (p< 0.01) are shown with odds ratios for each cohort (heatmap) and log transformed corrected p-value (barplot).

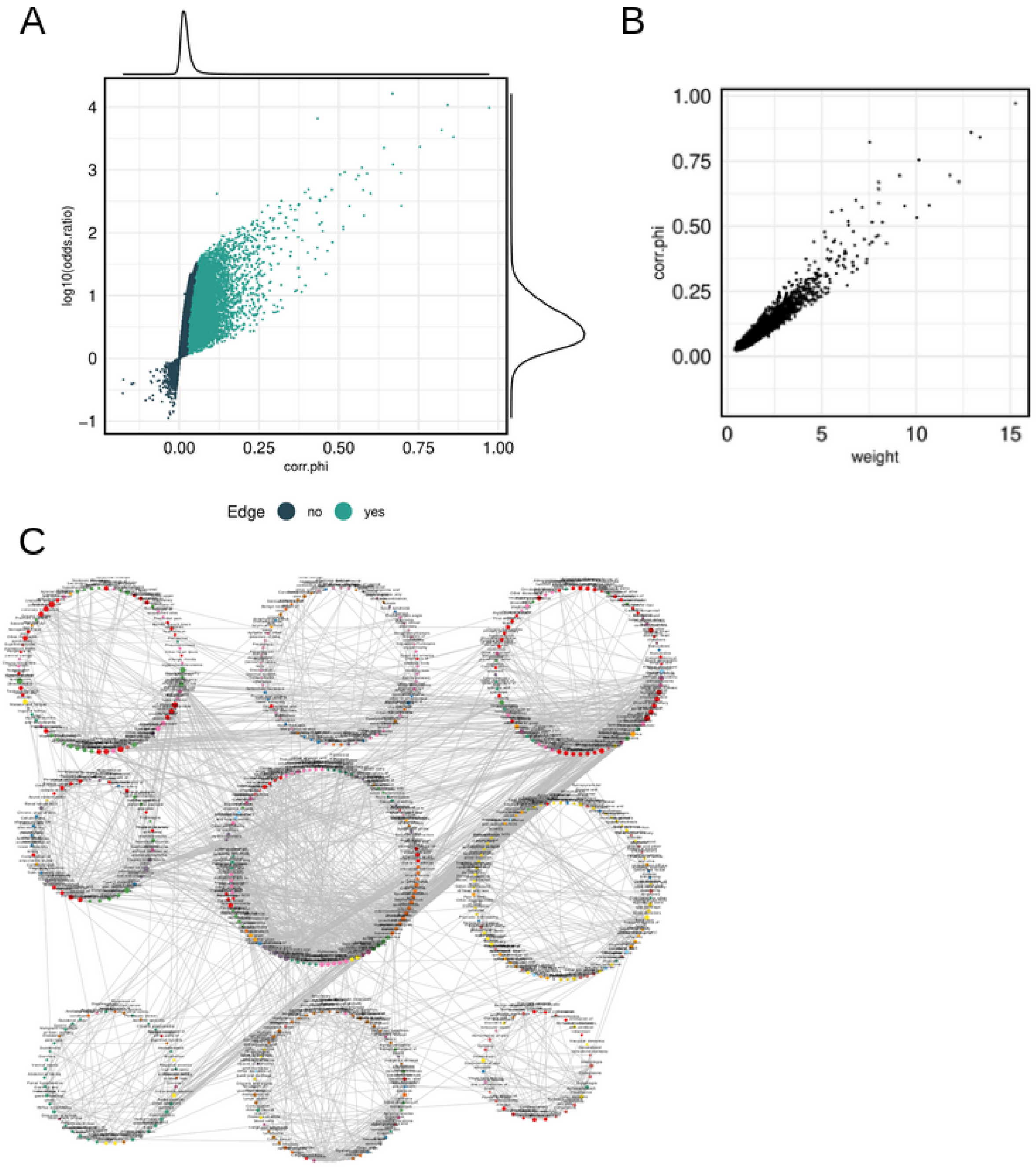

Fig. S6. HFnet overview.
A) Phi correlation and log odds ratio of pairwise disease comparison in the general HF cohort. B) Adjustment of phi-correlation. Correlation coefficients were scaled by disease. As low prevalence diseases are expected to result in lower phi-correlation coefficients and scaling by mean coefficient can address this effect. The calculated weights were used as edge weights in the HFnet. C) HFnet plotted and clustered by disease cluster (DC). DCs are arranged in rows (DC1, DC2, DC3..).

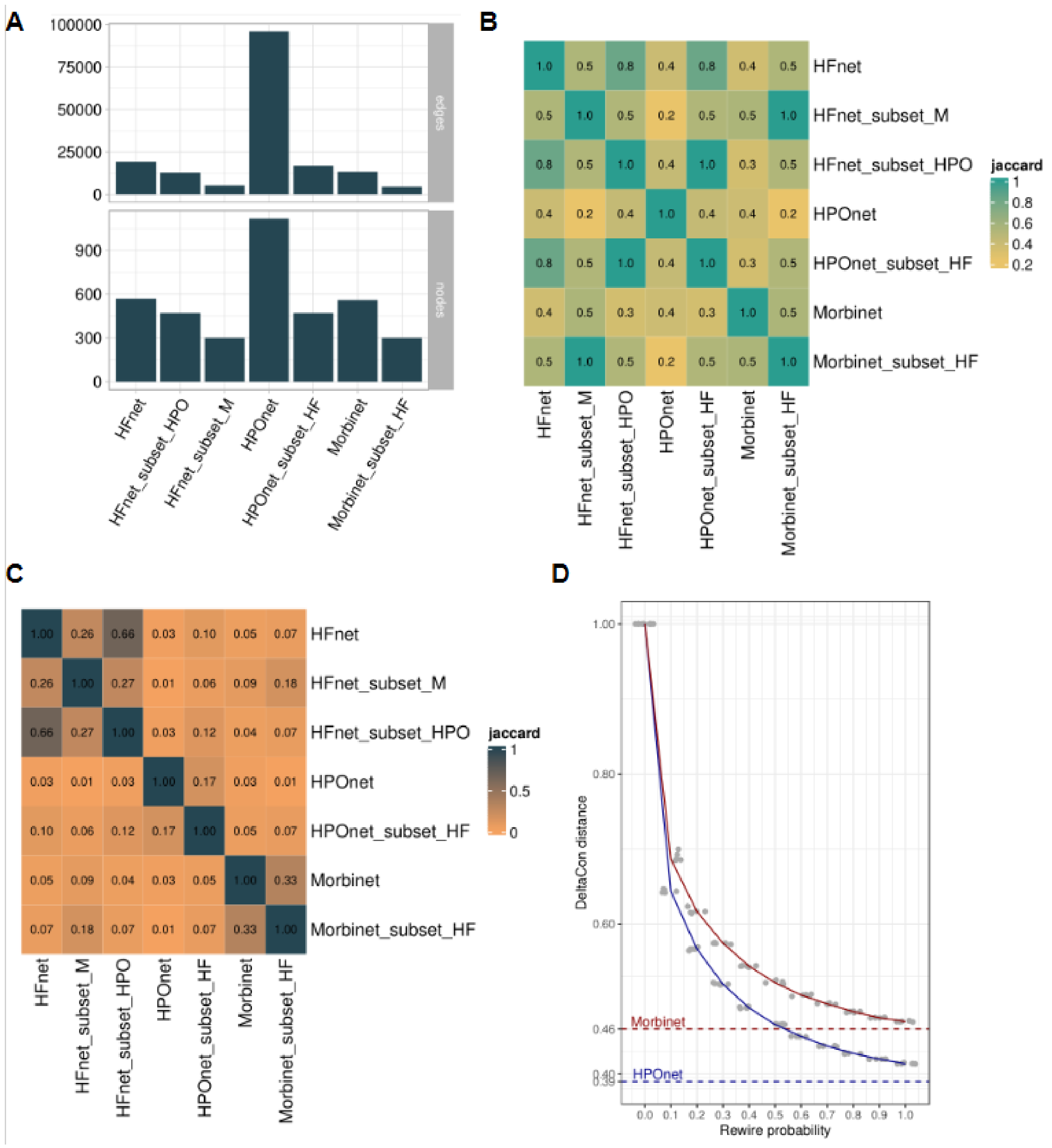

Fig. S7. Comparison of disease networks.

A) We compared the HFnet with two other disease networks. Morbinet represents an epidemiological comorbidity network from a larger and non HF centered cohort. HPOnet is a disease network based on ontological similarity of phenotypes. Both networks were initially larger than the HFnet, thus we subset each network to the same nodes. We compared number of edges (top panel) and number of edges (bottom panel). B) Jaccard comparison of nodes. C) Jaccard comparison of edges. D) DeltaCon similarity (y-axis) compared to rewiring probabilities of the HFnet (x-axis). We used subsets of the HFnet with Morbinet (red) and with HPOnet (blue) and rewired each subset five time with a given probability (x-axis) and computed DeltaCon similarity with the original HFnet. Dashed lines indicate HFnet and Morbinet and HFnet and HPOnet similarities.

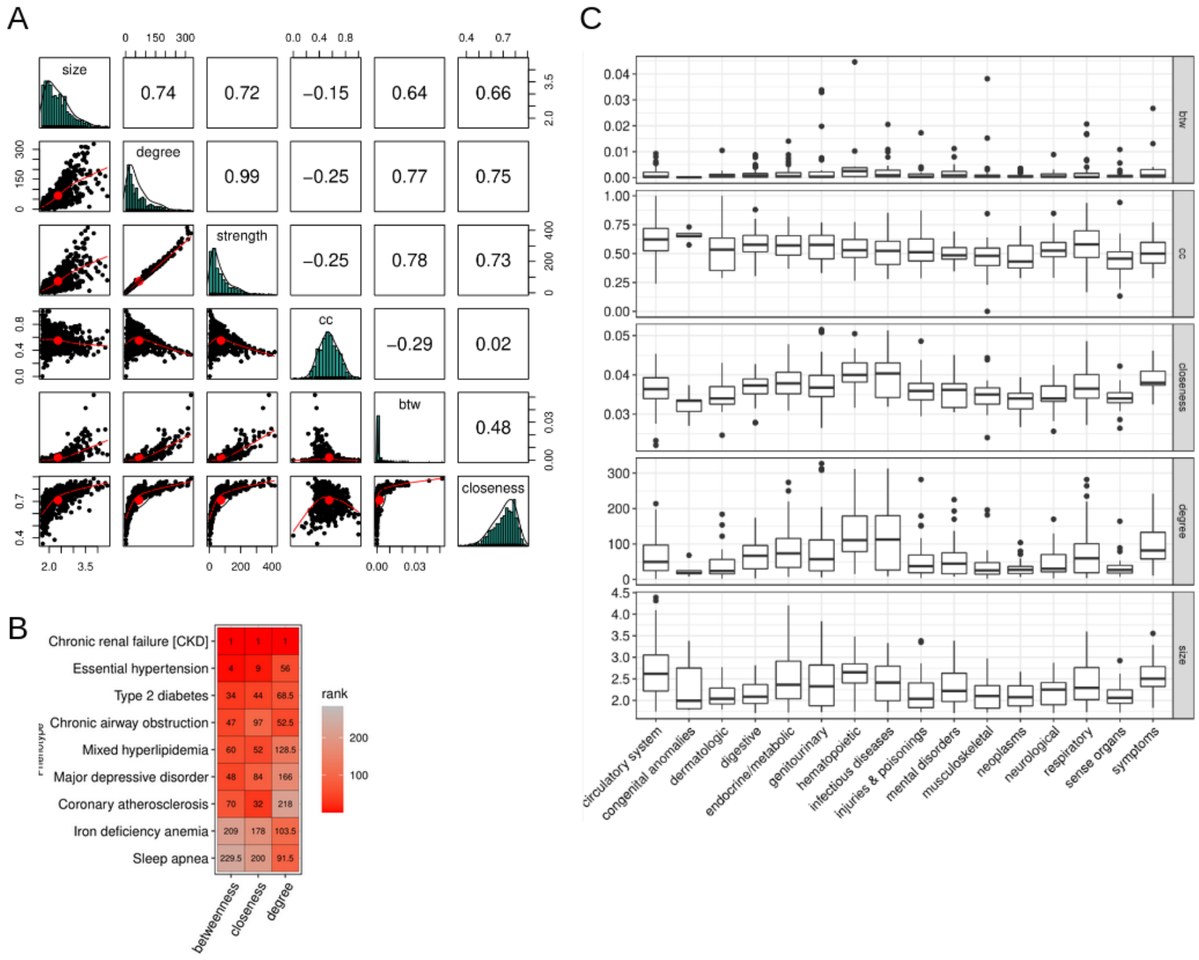

Fig. S8. Comparison of centralities.
A) Comparison of local (node-wise) graph theory based metrics. Size, log10(prevalence) per disease; degree, number of edges per disease; strength, sum of edge weights per disease, cc, cluster coefficient, number of connected vs unconnected first order neighbors per disease; btw, betweenness centrality (fraction of shortest paths with the node vs without the node); closeness, closeness centrality (inverse of the sum of distances to all the other vertices in the graph). Upper half displays Pearson's correlation between metrics.

B) Important comorbidities of HF compared via centrality rankings in the HFnet. C) Graph metrics compared by disease category in the HFnet. Kruskal.Wilis test $p < 0.01$ for all metrics except betweenness centrality (btw). Size, log10(prevalence); degree, number of edges; cc, cluster coefficient, number of connected vs unconnected first order neighbors; btw, betweenness centrality (fraction of shortest paths with the node vs. without the node); closeness, closeness centrality (inverse of the sum of distances to all the other vertices in the graph).

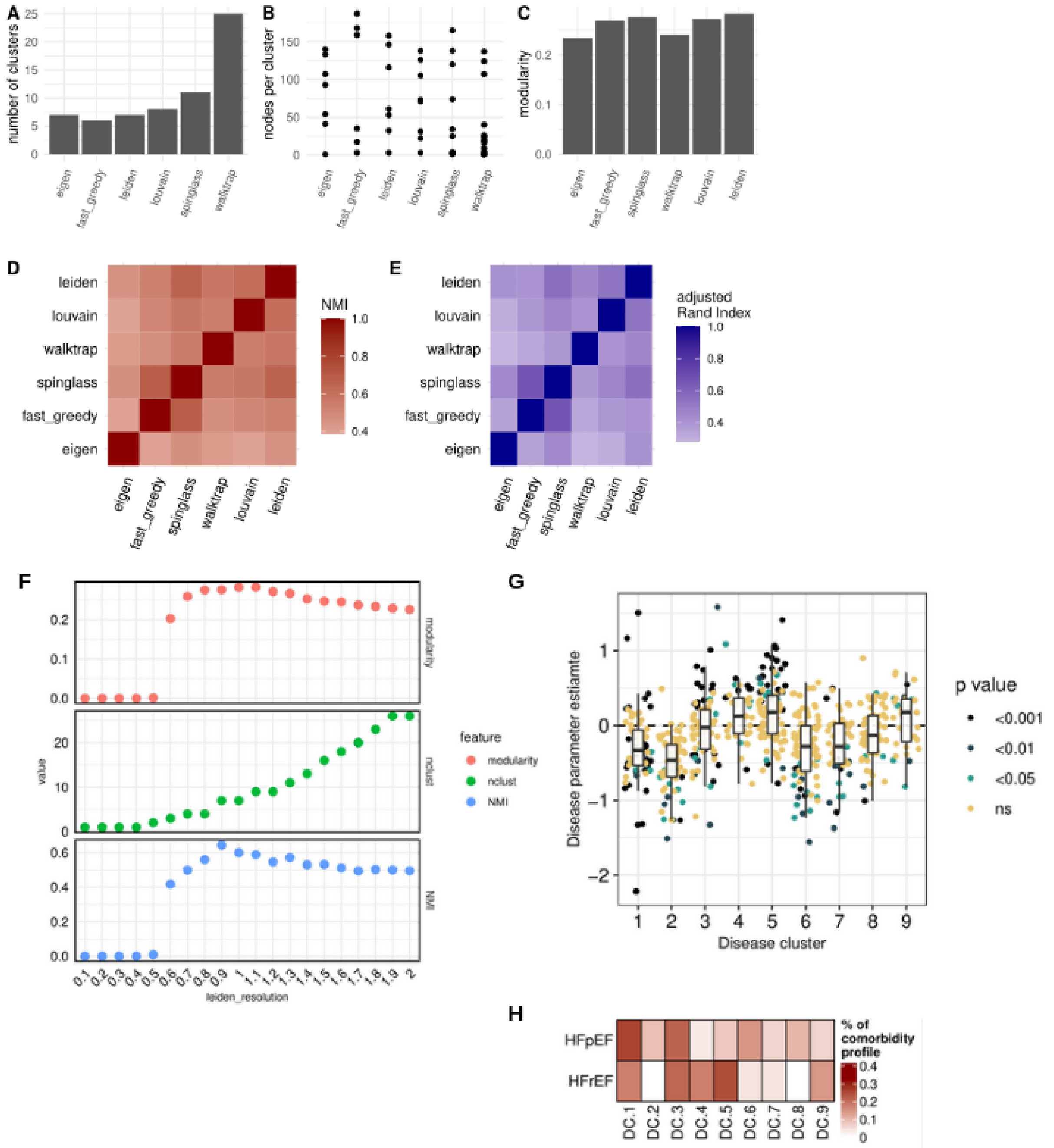

Fig. S9. Comparison of clustering algorithms in the HFnet.
A) Comparison of number of clusters by algorithms. B) Number of nodes per cluster. C) Network modularity achieved by algorithm. D&E) Comparison of similarity of node assignment between clustering algorithms with normalized mutual information (D) and adjusted rand index (E). F) Comparison of modularity , cluster number and mean normalized mutual information (with other algorithms) by resolution parameter in leiden algorithm. G) Comparisons of single disease parameters from logistic regression models for HFpEF/HFrEF contrast that were controlled for sex. Disease parameter estimates are on y-axis and significance of the parameter is color coded. H) Composition of the comorbidity profiles (rows) in disease clusters (DCs).

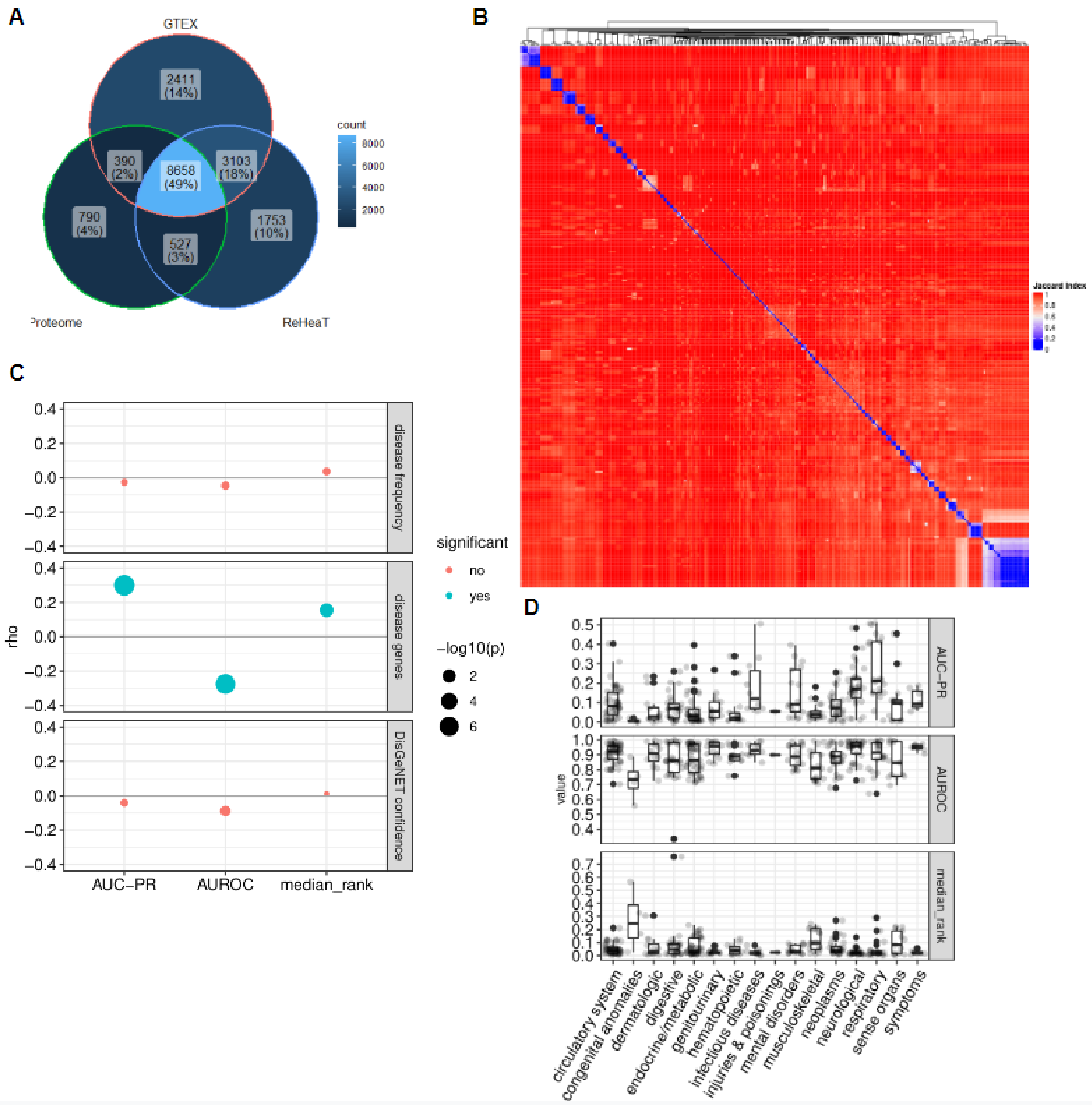

Fig. S10. Leave-one-out cross validation of disease gene prediction.
A) Genes expressed in the heart based on three data sources providing different levels of evidence. GTEX is used to describe gene expression in the human healthy heart, proteome study for expression on protein level in human heart and gene expression in diseased heart. We subset the gene networks to the union. B) Jaccard distance calculated between all DisGeNET gene sets linked to the HFnet. The blue clusters indicate similar gene sets, which were excluded during the leave one out cross validation assessment to avoid overfitting to DisGeNET. C) Correlation of link prediction with disease frequency (upper panel) and number of predicted genes (middle panel) and DisGeNET confidence scores (lower panel). D) Prediction metrics compared by disease categories

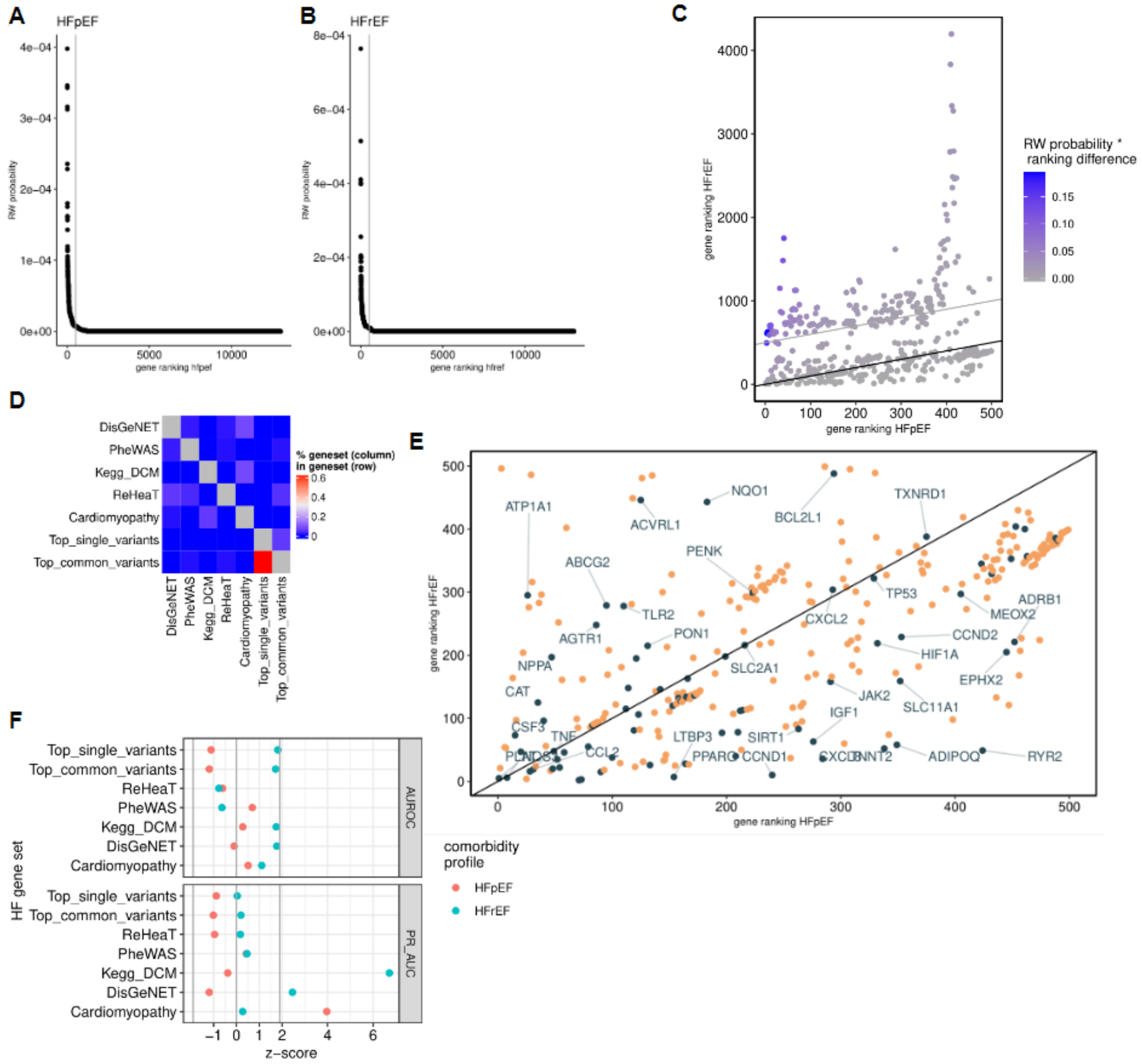

Fig. S11. HF subtype gene prediction.

A+B) Random walk (RW) with restart in multiplex heterogeneous network was applied with comorbidity profiles for HFpEF and HFrEF as seed nodes. RW probability distribution of all genes in the HFhetnet is shown for A) HFpEF and B), HFrEF comorbidity profile. C) Gene prioritization for HFpEF. Top 500 HFpEF genes are ranked (x-axis) and compared to their ranking in the HFrEF vector (y-axis). Color is the RW probability for HFpEF multiplied by the ranking difference. This calculation yields a new gene ranking that prioritizes HFpEF specific genes. D) Comparison of gene rankings within the top 500 genes of HFpEF (x-axis) and HFrEF (y-axis). Genes that are known to be associated with heart failure are colored and labeled. E) Comparison of intersections of HF gene sets demonstrating a low redundancy. F) HF geneset recovery (assessed with area under the receiver operator (AUROC) and area under the precision-recall curve (PR_AUC)) with 1000 random comorbidity profiles from the HFnet were used to generate null distributions. The geneset recovery values from the real HFpEF and HFrEF comorbidity profiles were then z-transformed.

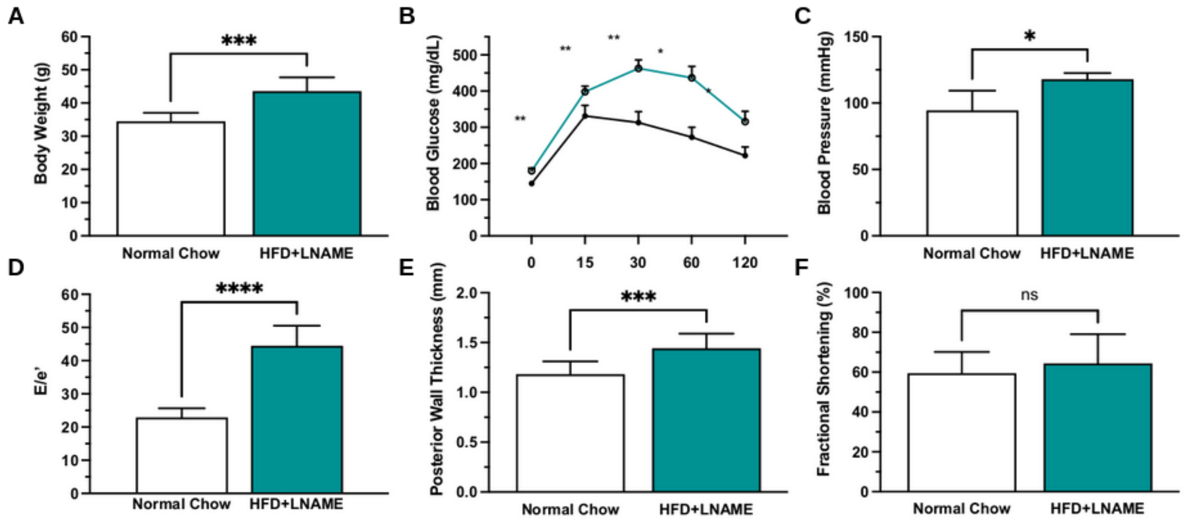

Fig. S12. L-NAME/HFD Phenotype data.
A) Body weight (g). B) Blood glucose levels (mg/dL). C) Blood Pressure (mmHg). D) Echocardiography based E/e'. E) Posterior wall thickness (mm), F) Fractional shortening (%). Unpaired, two sided student's t-test for all comparisons; * $p<0.05$, ** $p<0.01$, *** $p<0.001$.

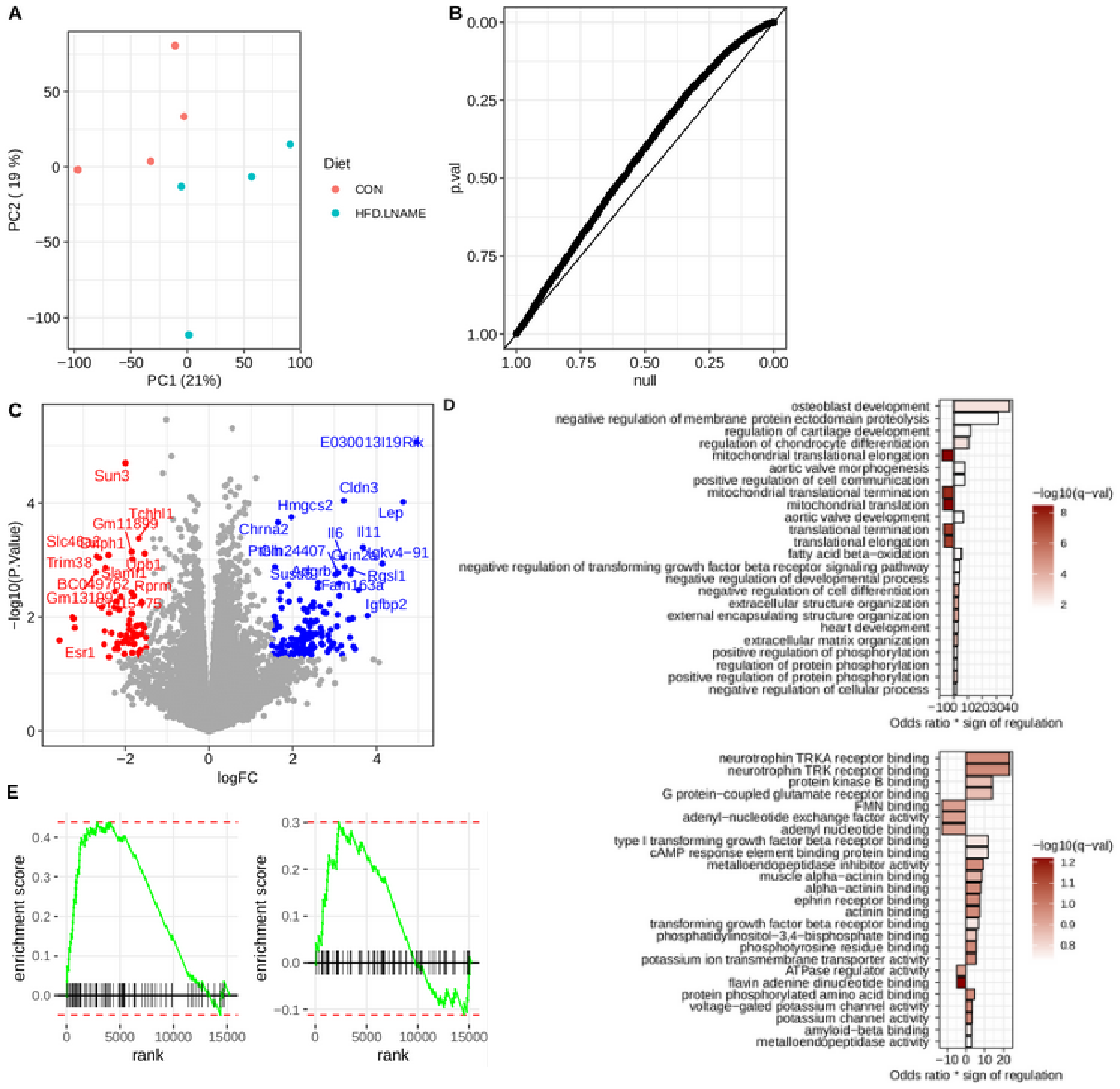

Fig. S13. Myocardial gene expression in L-NAME/HFD.
A) Principal component analysis embedding transcriptomes of murine control (CON) and HFpEF samples (HFD.LNAME). B) Q-Q plot displaying deviation of gene-level p-values from a null model. C) Volcanoplot displaying up and down regulated genes. D) Overrepresentation analysis of GO terms, upper panel GO biological process, lower panel GO molecular function. Enrichment was performed by selecting up and down regulated genes and enriching them separately by calculating Odds Ratio and hypergeometric tests within ontology gene sets. For visualization purposes we multiplied the Odds Ratio by sign of regulation. E) Visualization of the running sum calculated in gene set enrichment analysis for the comorbidity predicted gene sets. Left panel are top 100 HFpEF predicted genes, right panel are top 100 HFrEF predicted genes. Gene ranks were ordered by t-statistic values.
